# An interpretable XGBoost model for contrast-associated acute kidney injury in patients with acute coronary syndrome: development and geographical external validation

**DOI:** 10.3389/fdgth.2026.1917647

**Published:** 2026-08-18

**Authors:** Chuanwei Zhao, Honglin Li, Yane Yang, Ying Yang, Xinhua Wu

**Affiliations:** 1 Department of Cardiology, The First Affiliated Hospital of Dali University, Dali, Yunnan, China; 2 Department of Cardiology, The Second People’s Hospital of Baoshan City Yunnan Province, Baoshan, Yunnan, China; 3 Department of General Practice, People’s Hospital of Tengchong City, Tengchong, Yunnan, China

**Keywords:** acute coronary syndrome, clinical prediction model, contrast-associated acute kidney injury, coronary angiography, geographical external validation, machine learning, SHAP, XGBoost

## Abstract

**Background:**

Contrast-associated acute kidney injury (CA-AKI) is a clinically relevant complication after coronary angiography in patients with acute coronary syndrome (ACS). We aimed to develop and geographically validate a compact, interpretable machine-learning model and to determine whether the nonlinear algorithm provided incremental value beyond conventional regression using the same predictors.

**Methods:**

This retrospective two-center prediction study included patients with ACS undergoing coronary angiography. The derivation cohort was obtained from Dali, and the Baoshan cohort was reserved for locked geographical external validation. The primary XGBoost model used eight fixed predictors: age, blood urea nitrogen, baseline serum creatinine, fibrinogen, lymphocyte percentage, renin–angiotensin system inhibitor use, uric acid, and glycated hemoglobin A1c. Internal validation used nested five-fold cross-validation after establishment of the predictor set with fold-specific preprocessing. Performance was assessed using discrimination, calibration, prediction error, decision curve analysis, exploratory risk stratification, sensitivity analyses, and SHAP-based interpretation.

**Results:**

The derivation cohort included 2,677 patients, of whom 302 developed CA-AKI, and the external validation cohort included 1,539 patients, of whom 243 developed CA-AKI. The fixed eight-predictor XGBoost model achieved an internal out-of-fold area under the receiver operating characteristic curve (AUROC) of 0.876 and an external AUROC of 0.819. In external validation, the area under the precision–recall curve was 0.581, the Brier score was 0.096, the calibration slope was 0.747, and the observed-to-expected event ratio was 1.059. XGBoost substantially outperformed models based on baseline serum creatinine, estimated glomerular filtration rate, or basic clinical variables. However, its improvement over logistic regression using the same eight predictors was modest. Risk categories preserved clear risk ordering across hospitals, although lower-risk patients were underpredicted. Reduced-predictor models retained similar external performance, whereas complete-case analyses were limited by substantially smaller sample sizes.

**Conclusions:**

The fixed eight-predictor XGBoost model provided useful cross-hospital risk discrimination for CA-AKI, but its incremental advantage over same-variable logistic regression was limited and external calibration was imperfect. Prospective multicenter validation, standardized predictor timing, and local calibration assessment are required before clinical implementation.

## 1 Introduction

Contrast-associated acute kidney injury (CA-AKI) remains an important complication after coronary angiography and percutaneous coronary intervention, particularly in patients with acute coronary syndrome (ACS), who frequently undergo urgent invasive evaluation in the setting of hemodynamic and metabolic stress (1). Post-procedural acute kidney injury is associated with adverse short- and long-term outcomes, including increased mortality, making early risk assessment clinically relevant even when the direct contribution of contrast media cannot be isolated with certainty (2). Current acute kidney injury definitions rely primarily on changes in serum creatinine (3), while updated European guidance emphasizes that kidney injury occurring after contrast exposure may be multifactorial and should be interpreted as a temporal association rather than automatic proof of contrast toxicity (4). Contemporary consensus statements similarly recommend balancing renal risk assessment against the potential harm of delaying clinically indicated imaging or intervention (5).

Risk prediction after coronary angiography has traditionally relied on clinical scores. The original Mehran score combined baseline clinical and procedural characteristics to estimate contrast-related kidney injury after percutaneous coronary intervention (6), and a contemporary score was subsequently developed to reflect changes in patient profiles and interventional practice (2). However, a systematic review found substantial heterogeneity among available models in outcome definitions, predictor selection, handling of missing data, and validation methods (7). Several investigators have therefore focused on preprocedural models that can be applied before contrast administration (8). Machine-learning approaches have also been evaluated in acute myocardial infarction and broader coronary angiography populations, with studies reporting potentially useful discrimination from routinely collected clinical variables (9, 10). More recently, a model based specifically on pre-catheterization variables further illustrated the feasibility of data-driven prediction without relying on information generated during the procedure (11).

Important uncertainties nevertheless remain. Many established scores depend on procedural variables that are unavailable when the initial risk decision is made, whereas preprocedural models may lose information that becomes clinically relevant during an urgent ACS presentation. Published machine-learning studies have used different predictor sets and validation designs, limiting direct comparison and increasing the possibility of optimistic performance estimates (10). Differences in preprocessing, hyperparameter tuning, and model comparison can further influence reported performance (12). External validation has often been limited, and discrimination has sometimes been emphasized without equally detailed evaluation of calibration, prediction error, or decision-analytic utility. It is also frequently unclear whether apparent improvement reflects the machine-learning algorithm itself or simply the inclusion of a more informative predictor set, because comparisons with conventional regression using exactly the same variables are uncommon. In addition, predictor availability and measurement practices can differ materially between hospitals, while complex model outputs require interpretable analyses that describe how individual variables contribute to predictions without implying causality. These issues are especially relevant for compact electronic health record models intended for cross-hospital use in patients with ACS.

Accordingly, we conducted a retrospective two-center prediction study among patients with ACS undergoing coronary angiography. We aimed to develop and geographically validate an interpretable XGBoost model based on a fixed set of eight routinely recorded clinical and laboratory predictors, and to compare its performance with simple clinical benchmarks and logistic regression using the same predictors. The central question was whether this compact predictor set could provide transportable risk estimation across hospitals and whether a nonlinear machine-learning algorithm added information beyond conventional modeling of the same variables. We further evaluated discrimination, calibration, prediction error, decision-analytic utility, exploratory risk stratification, predictor availability, and model interpretability under a locked external-validation framework.

## 2 Methods

### 2.1 Ethics statement

This retrospective study was approved by the Ethics Committee of The Second People's Hospital of Baoshan City, Yunnan Province, China [approval number 2025-032(other)-01]. The requirement for written informed consent was waived because the study used routinely collected retrospective data. Direct identifiers were removed before analysis, and no directly identifying information was included in the modeling datasets. The study was conducted in accordance with the Declaration of Helsinki.

### 2.2 Study design and reporting framework

**Figure 1:**
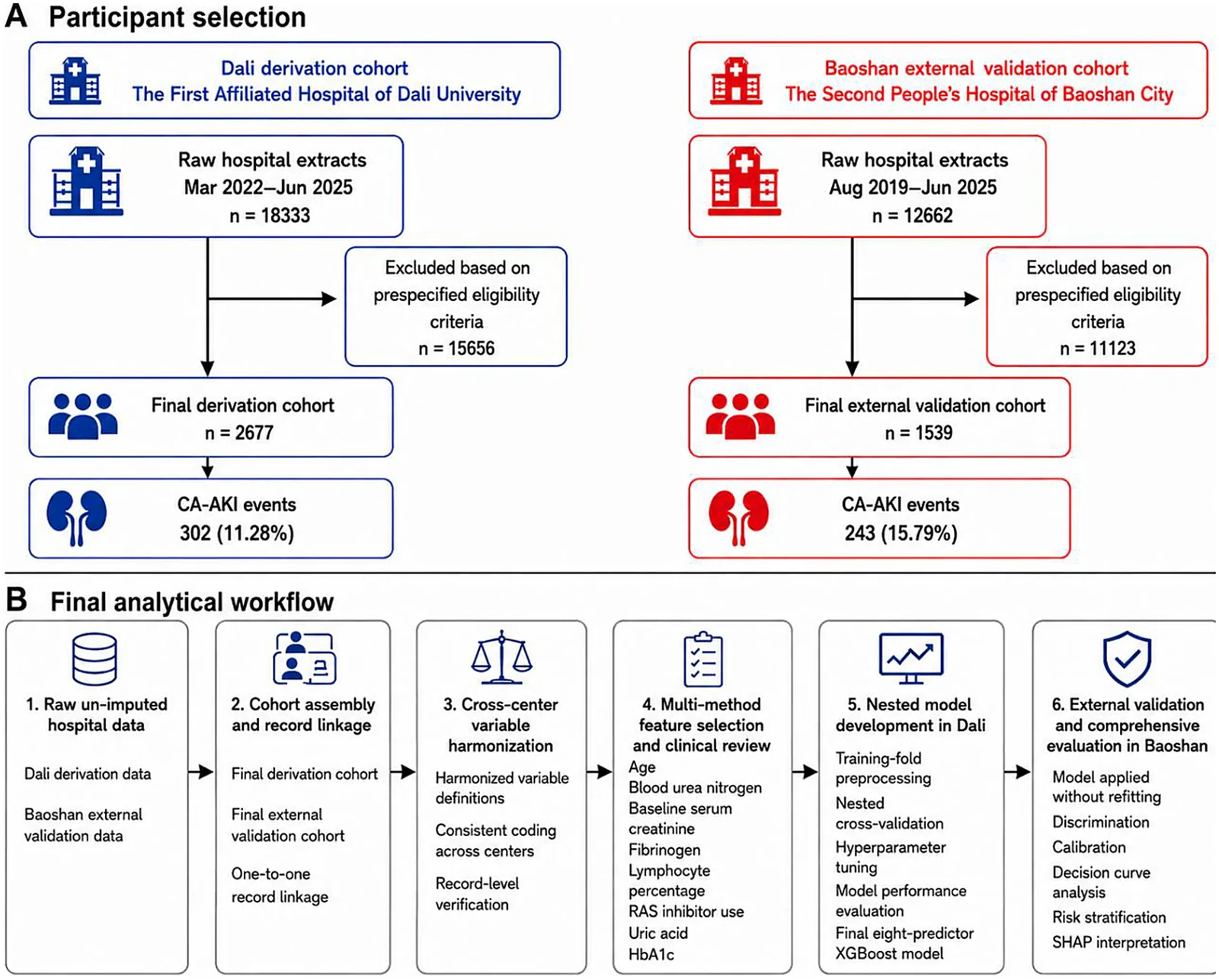
Participant selection and analytical workflow for development and geographical external validation of the CA-AKI prediction model. **(A)** Participant selection in the Dali derivation cohort and the Baoshan geographical external validation cohort. The final derivation cohort included 2,677 patients, of whom 302 developed CA-AKI, and the external validation cohort included 1,539 patients, of whom 243 developed CA-AKI. **(B)** Analytical workflow comprising construction of the raw unimputed hospital datasets, cohort assembly and record linkage, cross-center variable harmonization, multi-method feature selection and clinical review in the Dali derivation cohort, nested model development in Dali, and locked geographical external validation in Baoshan without model refitting. The final evaluation included discrimination, calibration, decision curve analysis, exploratory risk stratification, and SHAP-based model interpretation. CA-AKI, contrast-associated acute kidney injury; SHAP, SHapley Additive exPlanations.

This was a retrospective, two-center clinical prediction model study involving model development, nested internal validation, and geographical external validation. The derivation cohort was obtained from The First Affiliated Hospital of Dali University, and the geographical external validation cohort was obtained from The Second People's Hospital of Baoshan City. The analytical workflow was organized according to contemporary principles for transparent reporting and assessment of clinical prediction models using regression or machine learning methods (13, 14). The Baoshan cohort was reserved for a single locked external evaluation and was not used for predictor selection, preprocessing decisions, hyperparameter tuning, threshold selection, model selection, or recalibration. An independent temporal validation analysis was not performed because the available analytical datasets did not include the patient-level dates required to construct and verify a chronological validation cohort. The final study workflow is summarized in Figure 1.

### 2.3 Study setting, participants, and data sources

The Dali derivation cohort included hospitalizations between March 2022 and June 2025, and the Baoshan external validation cohort included hospitalizations between August 2019 and June 2025. A total of 18,333 hospital records were screened in Dali and 12,662 in Baoshan. Eligible patients were hospitalized with acute coronary syndrome (ACS) who underwent coronary angiography during the index hospitalization and had the creatinine fields required to derive the study outcome. Patients receiving maintenance hemodialysis were excluded. Cohort membership was determined from the finalized clinical cohort linkage and eligibility records. After application of the prespecified criteria, 2,677 patients were included in the Dali derivation cohort and 1,539 in the Baoshan geographical external validation cohort. All eligible patients were included; no formal *a priori* sample-size calculation was used.

Clinical variables were extracted from the electronic medical record and laboratory information systems of the participating hospitals. The available domains included demographics, cardiovascular risk factors, comorbidities, admission characteristics, laboratory measurements, echocardiographic variables, procedure-related information, and medication records. ACS eligibility was based on the final clinical or discharge diagnosis recorded by the treating cardiology team in the electronic medical record. No independent central adjudication of ACS subtype was performed. ACS presentation could be classified reliably as ST-segment elevation myocardial infarction or non-ST-segment elevation ACS. The source data did not support a reliable separation of non-ST-segment elevation myocardial infarction from unstable angina; therefore, these patients were retained in a combined non-ST-segment elevation ACS category.

### 2.4 Outcome definition

The primary outcome was contrast-associated acute kidney injury (CA-AKI). In the harmonized analytical datasets, CA-AKI was operationalized using the serum creatinine component of the Kidney Disease: Improving Global Outcomes and European Society of Urogenital Radiology definitions: an increase in serum creatinine of at least 26.5 μmol/L or an increase to at least 1.5 times the value designated as baseline (3, 4). During cohort construction, coronary angiography timestamps and serum creatinine specimen-collection timestamps were used to identify the most recent serum creatinine measurement obtained before coronary angiography during the index hospitalization as baseline; no additional maximum preprocedural interval was imposed. The maximum serum creatinine value obtained 48–72 h after angiography was used as the follow-up value. These timestamp variables were used for outcome ascertainment but were not included as predictors in the final modeling dataset.

### 2.5 Predictor definitions and cross-center harmonization

The candidate-predictor inventory was based on clinical relevance, routine availability in the participating electronic health record systems, and availability in the finalized analytical extracts. A common cross-center data dictionary was applied to harmonize variable names, coding, units, and category labels (15). Continuous variables were converted to numeric format and checked for implausible values and incompatible scales. Binary variables were encoded as 0/1 indicators, and multicategory variables were represented using indicator coding when required by a model. Center-specific availability and missingness were retained rather than being treated as complete data. Definitions, units, coding, and missingness of the fixed predictors are reported in Supplementary Table S1, and the full candidate-variable missingness audit is reported in Supplementary Table S2.

Except for the serum creatinine measurements used to define CA-AKI, exact measurement times relative to coronary angiography could not be consistently verified for the other laboratory and medication predictors across the two hospital systems. These predictors were obtained from the available admission or baseline analytical records and were not treated as measurements verified to occur within a precise preprocedural window. No predictor was derived from follow-up creatinine measurements, absolute or relative creatinine changes, derived CA-AKI indicators, or other outcome-defining information. The estimated glomerular filtration rate (eGFR) was calculated using the 2021 CKD-EPI race-free creatinine equation after conversion of serum creatinine from μmol/L to mg/dL (16). Renin–angiotensin system inhibitor use included angiotensin-converting enzyme inhibitors, angiotensin receptor blockers, and angiotensin receptor–neprilysin inhibitors.

### 2.6 Feature selection, fixed predictor set, and comparator models

The fixed predictor set was established through a feature-selection framework followed by clinical review. Candidate predictors were first screened for redundancy and multicollinearity using pairwise correlation assessment and variance inflation factor analysis. Four complementary feature-selection approaches, including Boruta, least absolute shrinkage and selection operator (LASSO) logistic regression, recursive feature elimination with random forest (RF-RFE), and genetic algorithm-based selection, were applied in the derivation cohort. Predictors supported by at least three of the four approaches were considered eligible for retention. The final predictor set was determined after reviewing clinical interpretability, availability in the admission or baseline analytical extracts, cross-center consistency, and the potential risk of outcome leakage.

**Figure 2:**
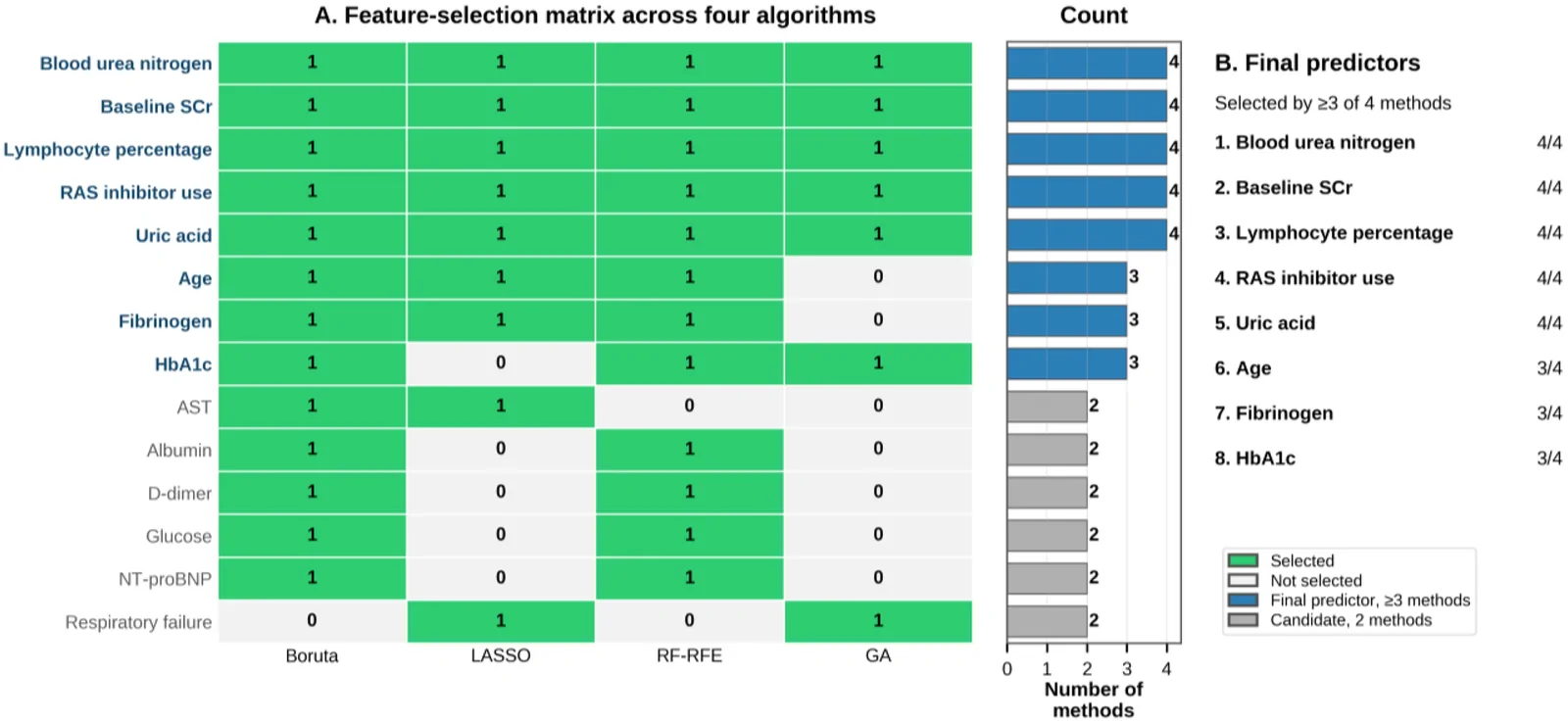
Feature selection across four algorithms and final predictors. **(A)** Feature-selection matrix showing whether each candidate predictor was selected by Boruta, LASSO logistic regression, RF-RFE, and genetic algorithm. The adjacent bar plot indicates the number of methods selecting each predictor. **(B)** Final predictors retained for model development, defined as variables selected by at least three of the four feature-selection methods. LASSO, least absolute shrinkage and selection operator; RF-RFE, random forest-based recursive feature elimination; GA, genetic algorithm; SCr, serum creatinine; RAS, renin–angiotensin system; HbA1c, glycated hemoglobin A1c; AST, aspartate aminotransferase; NT-proBNP, N-terminal pro-B-type natriuretic peptide.

The resulting fixed eight-predictor set consisted of age, blood urea nitrogen, baseline serum creatinine, fibrinogen, lymphocyte percentage, renin–angiotensin system inhibitor use, uric acid, and glycated hemoglobin A1c (HbA1c). These variables represented age-related vulnerability, renal function, inflammatory and coagulation status, metabolic risk, and medication exposure recorded in the admission or baseline analytical extracts. No predictor was derived from follow-up creatinine measurements or other outcome-defining information. The eight-variable set was locked before model comparison and was applied unchanged during internal validation and geographical external validation (Figure 2).

The primary model was an extreme gradient boosting (XGBoost) classifier using the fixed eight predictors (17). Four principal benchmark models were evaluated under the same validation framework: a logistic regression model using baseline serum creatinine alone, a logistic regression model using eGFR alone, a basic clinical logistic regression model containing age, sex, diabetes mellitus, and eGFR, and logistic regression using the same fixed eight predictors as XGBoost. The same-variable logistic model was included to separate the contribution of the predictor set from the incremental contribution of the nonlinear algorithm.

A standardized comparison of 15 candidate algorithms was also performed using the fixed eight predictors: XGBoost, generalized additive model, Gaussian process classifier, gradient boosting decision tree, random forest, support vector machine, naive Bayes, LightGBM, rotation forest, logistic regression, AdaBoost, extremely randomized trees, multilayer perceptron, k-nearest neighbors, and decision tree. This comparison was used to describe metric-dependent variation across algorithms and was not used to replace the primary model on the basis of external performance. No composite AB-score was used for model selection. Model definitions, search spaces, and final hyperparameters are provided in Supplementary Tables S3, S4.

The feasibility of calculating the original Mehran and Mehran 2 scores was assessed separately. Reliable calculation was not possible because essential variables, including contrast volume, hypotension, intra-aortic balloon pump use, and several contemporary procedural variables, were unavailable or not equivalent in definition. Approximate scores were therefore not constructed, and no unvalidated substitutions were made (Supplementary Table S5).

### 2.7 Missing data and preprocessing

Missingness was quantified before imputation at both the variable and patient levels. The primary analysis used all eligible patients rather than restricting model development to complete cases. Within each outer cross-validation training fold, missing continuous and binary predictor values were imputed using medians estimated only from that training fold. The fitted imputation values were then applied to the corresponding outer validation fold. Variables were standardized within the training fold for algorithms requiring scaling, including logistic regression, support vector machines, k-nearest neighbors, multilayer perceptrons, Gaussian process classifiers, generalized additive models, and rotation forest. Tree-based algorithms were not standardized unless required by their implementation.

After completion of internal validation and hyperparameter selection, each locked model was refitted using the full Dali cohort. Imputation and scaling parameters estimated from the full Dali cohort were then applied unchanged to the Baoshan cohort. No preprocessing parameter was estimated from the external cohort. HbA1c availability was specifically audited because of substantial between-center missingness. Complete-case, HbA1c-availability, and reduced-predictor sensitivity analyses were conducted to evaluate the dependence of performance on less consistently available laboratory variables.

### 2.8 Model development and nested internal validation

After the fixed eight-predictor set had been established in the Dali derivation cohort, model development and internal performance estimation were conducted using nested stratified cross-validation (18). The feature-selection procedure was not repeated within the cross-validation folds; therefore, the internal performance estimates were conditional on the previously established predictor set. The outer loop consisted of five stratified folds with shuffling and generated one out-of-fold prediction for every participant. Within each outer-training fold, a three-fold stratified grid search was used for hyperparameter tuning, with negative log loss as the optimization criterion. Median imputation, standardization when required by the algorithm, and hyperparameter selection were fitted exclusively within each outer-training fold and then applied to the corresponding outer validation fold. The Baoshan external validation cohort was not accessed during predictor selection, preprocessing, hyperparameter tuning, threshold selection, or internal performance estimation. A common random seed of 20,260,715 was used for reproducibility.

All internal performance estimates were calculated from the combined out-of-fold predictions rather than from predictions generated for patients used to fit the corresponding model. After completion of internal evaluation, a final five-fold stratified grid search was performed within the complete Dali cohort to select the locked hyperparameters for each full-cohort model. Imputation and scaling parameters were then estimated from the complete Dali cohort, and the final estimator was fitted to all eligible Dali participants. The fitted model, preprocessing parameters, and hyperparameters were frozen before application to the Baoshan cohort. Full hyperparameter grids, selected parameters, software implementations, and random seeds are reported in Supplementary Table S4.

### 2.9 Geographical external validation

The frozen Dali models were applied once to the Baoshan cohort to assess geographical transportability (19). The predictor definitions, preprocessing rules, imputation values, scaling parameters, model coefficients or tree structures, classification thresholds, and risk-category cutoffs were transferred without refitting. No recalibration, center-specific hyperparameter tuning, threshold optimization, predictor reselection, or model selection was performed in Baoshan. The external cohort therefore provided a fully locked evaluation of model discrimination, calibration, prediction error, and clinical utility.

### 2.10 Performance measures and statistical inference

Continuous variables were summarized as medians with interquartile ranges, and categorical variables as counts and percentages. Between-cohort differences in baseline characteristics were summarized using absolute standardized mean differences rather than significance tests; values of at least 0.10 were considered potentially meaningful (20). Descriptive summaries were calculated from available observations, and missingness was reported separately.

Discrimination was assessed using the area under the receiver operating characteristic curve (AUROC) and the area under the precision–recall curve (AUPRC). Overall prediction error was quantified using the Brier score and log loss (21). The scaled Brier score was calculated as 1 minus the ratio of the model Brier score to the null-model Brier score, where the null-model Brier score was prevalence × (1−prevalence). Calibration was assessed using calibration-in-the-large, calibration slope, the observed-to-expected event ratio (O/E), and calibration plots based on groups of predicted risk (22). Calibration-in-the-large was estimated using the logit of predicted risk as an offset, and the calibration slope was estimated by regressing the observed outcome on the logit of predicted risk.

Ninety-five percent confidence intervals for AUROC and AUPRC were obtained using 2,000 outcome-stratified bootstrap resamples. Differences between the primary XGBoost model and comparator models in AUROC, AUPRC, and Brier score were evaluated using 2,000 paired outcome-stratified bootstrap resamples so that predictions for the same participants remained paired. Two-sided bootstrap *P* values were reported for paired AUROC differences. Confidence intervals for O/E ratios were derived from exact Poisson limits for the observed event count. The classification threshold for each model was selected from the Dali out-of-fold predictions using the Youden index and was then applied unchanged to Baoshan. Sensitivity, specificity, positive predictive value, negative predictive value, F1 score, and accuracy were calculated at the locked threshold.

### 2.11 Decision curve analysis and exploratory risk stratification

Decision curve analysis was used to compare net benefit across threshold probabilities from 0.01 to 0.50 (23, 24). The primary XGBoost model was compared with the principal benchmark models and with the treat-all and treat-none strategies. Decision curves were interpreted jointly with discrimination and calibration and were not used as evidence of a unique clinical advantage when net-benefit differences between the fixed eight-predictor XGBoost and same-variable logistic models were small.

Exploratory model-based risk categories were defined using the 50th, 75th, and 90th percentiles of the fixed eight-predictor XGBoost out-of-fold probability distribution in the Dali cohort. These cutoffs defined low-, intermediate-, high-, and very-high-risk groups and were fixed before application to Baoshan. For each group, the number of patients, number of events, observed event rate with an exact binomial confidence interval, mean predicted risk, and O/E ratio with an exact Poisson confidence interval were reported. These categories were intended to evaluate risk ordering and transportability and were not treated as guideline-endorsed intervention thresholds.

### 2.12 Sensitivity and age-stratified analyses

Three reduced-predictor strategies were evaluated using both logistic regression and XGBoost under the same nested-validation and locked-external-validation framework: a seven-predictor model excluding HbA1c; a five-predictor model excluding HbA1c, fibrinogen, and uric acid; and a Routine4 model containing age, blood urea nitrogen, baseline serum creatinine, and lymphocyte percentage. These analyses assessed whether model performance was maintained when less consistently available laboratory variables were omitted. Complete-case analyses were performed among patients with all eight predictors observed. HbA1c-measured and HbA1c-missing groups were also compared descriptively to assess potential informative availability. Sensitivity-analysis specifications are reported in Supplementary Tables S6, S7.

A targeted age-stratified evaluation was performed using prespecified groups of less than 60 years and at least 60 years. The overall locked model, preprocessing procedure, and Dali-derived classification threshold were applied without subgroup-specific refitting, recalibration, or threshold optimization. Within each age group, AUROC, AUPRC, Brier score, scaled Brier score, calibration slope, O/E ratio, and classification metrics were calculated. The difference in AUROC between age groups was evaluated using 2,000 outcome-stratified bootstrap resamples within each group. One Baoshan participant with missing age was excluded only from the age-stratified analysis; the participant remained in the overall external validation through the prespecified preprocessing pipeline. The age analysis was considered targeted validation rather than a broad multiple-subgroup search (Supplementary Table S8 and Supplementary Figure S1).

### 2.13 Model interpretation

The final fixed eight-predictor XGBoost model fitted to the complete Dali cohort was interpreted using exact TreeSHAP contributions generated by the native XGBoost prediction routine (25). Global feature importance was summarized using the mean absolute SHAP value. A SHAP summary plot displayed the direction and magnitude of patient-level contributions, and dependence plots displayed each predictor value against its corresponding SHAP value (26). SHAP values were expressed on the model log-odds scale. Three representative local explanations were selected using the Dali out-of-fold risk distribution: a non-event near the 10th percentile of non-event risk, a participant near the overall median risk, and an event near the 90th percentile of event risk. The locked full-cohort model was then used to display the feature contributions for these selected participants.

Partial dependence plots were generated from the Dali data after application of the final Dali-fitted median imputation values (27). For continuous predictors, model predictions were averaged after setting the predictor to 25 grid values spanning its 5th to 95th percentile range; the binary renin–angiotensin system inhibitor variable was evaluated at 0 and 1. SHAP and partial dependence analyses were used only to describe the behavior of the fitted prediction model. They were not interpreted as causal effects, exposure–response relationships, or validated clinical thresholds. The study did not include causal-inference analyses.

### 2.14 Software and reproducibility

Analyses were performed in Python 3.11.15 using numpy 1.26.4, pandas 2.3.3, scipy 1.16.3, scikit-learn 1.8.0, statsmodels 0.14.6, xgboost 3.2.0, lightgbm 4.6.0, and pygam 0.12.0. All primary results were generated from patient-level out-of-fold or locked external predictions and linked to analysis-grade source tables. Model definitions, tuning procedures, final hyperparameters, software versions, and seeds are reported in Supplementary Table S4. All tests were two-sided where applicable.

## 3 Results

### 3.1 Study population and baseline characteristics

After application of the eligibility criteria, 2,677 patients were included in the Dali derivation cohort and 1,539 in the Baoshan geographical external validation cohort (Figure 1). CA-AKI occurred in 302 patients (11.3%) in Dali and 243 patients (15.8%) in Baoshan. Compared with the derivation cohort, the external validation cohort was older (median age, 66.0 vs. 60.0 years), included a lower proportion of men (68.1% vs. 78.8%), and had a lower median body mass index (23.0 vs. 24.2 kg/m^2^). The proportion of patients with ST-segment elevation myocardial infarction was lower in Baoshan (33.7% vs. 65.1%), with a corresponding increase in non-ST-segment elevation ACS (66.3% vs. 34.9%). Baseline serum creatinine, blood urea nitrogen, uric acid, fibrinogen, hypertension, atrial fibrillation, and left ventricular ejection fraction were relatively similar between cohorts, whereas age, HbA1c, lymphocyte percentage, and renin–angiotensin system inhibitor use showed potentially meaningful between-center differences (Table 1 and Supplementary Table S9).

**Table 1:** Baseline characteristics of the derivation and geographical external validation cohorts.

Characteristic	Dali derivation cohort (*n* = 2,677)	Baoshan external validation cohort (*n* = 1,539)	SMD
Demographics			
Age, years	60.0 (53.0, 70.0)	66.0 (57.0, 73.0)	0.338
Male sex, *n* (%)	2,110 (78.8)	1,048 (68.1)	0.245
Body mass index, kg/m^2^	24.2 (23.3, 25.1)	23.0 (20.6, 25.4)	0.364
Current smoking, *n* (%)	1,250 (46.7)	523 (34.0)	0.261
Current alcohol use, *n* (%)	718 (26.8)	314 (20.4)	0.152
Clinical presentation and comorbidities			
STEMI, *n* (%)	1,743 (65.1)	518 (33.7)	0.663
NSTE-ACS, *n* (%)	934 (34.9)	1,021 (66.3)	0.663
Hypertension, *n* (%)	1,410 (52.7)	813 (52.8)	0.003
Diabetes mellitus, *n* (%)	624 (23.3)	288 (18.7)	0.113
Heart failure, *n* (%)	2,307 (86.2)	952 (61.9)	0.577
Atrial fibrillation, *n* (%)	115 (4.3)	75 (4.9)	0.028
Renal function and fixed model predictors			
Baseline serum creatinine, μmol/L	77.0 (66.0, 93.0)	75.3 (62.8, 92.5)	0.003
eGFR, mL/min/1.73 m^2^	93.5 (76.4, 103.2)	91.7 (73.0, 101.5)	0.129
< 30 mL/min/1.73 m^2^, *n* (%)	45 (1.7)	41 (2.7)	0.067
30–59 mL/min/1.73 m^2^, *n* (%)	235 (8.8)	186 (12.1)	0.108
60–89 mL/min/1.73 m^2^, *n* (%)	844 (31.5)	493 (32.0)	0.011
≥90 mL/min/1.73 m^2^, *n* (%)	1,553 (58.0)	818 (53.2)	0.098
Blood urea nitrogen, mmol/L	5.79 (4.61, 7.15)	5.81 (4.64, 7.46)	0.094
Uric acid, μmol/L	359.0 (294.0, 433.0)	346.0 (276.4, 434.5)	0.053
Glycated hemoglobin A1c, %	6.62 (6.13, 7.22)	6.00 (5.50, 7.60)	0.208
Fibrinogen, g/L	3.27 (2.75, 4.12)	3.33 (2.88, 3.95)	0.087
Lymphocyte percentage, %	22.5 (16.0, 28.8)	19.0 (12.6, 26.0)	0.313
Renin–angiotensin system inhibitor use, *n* (%)	816 (30.5)	668 (43.4)	0.270
Other clinical characteristics and outcome			
Left ventricular ejection fraction, %	65.0 (60.2, 70.0)	65.0 (58.0, 72.0)	0.061
CA-AKI, *n* (%)	302 (11.3)	243 (15.8)	0.132

Data are presented as median (interquartile range) for continuous variables and *n* (%) for categorical variables. Continuous variables were summarized among available observations; detailed feature-level missingness is reported in the Supplementary Materials. eGFR was calculated using the 2021 CKD-EPI race-free creatinine equation. NSTE-ACS includes NSTEMI and unstable angina because these subtypes could not be reliably distinguished in the source data. SMD values are presented as absolute values; an SMD ≥0.10 indicates a potentially meaningful between-cohort difference. ACS, acute coronary syndrome; BUN, blood urea nitrogen; CA-AKI, contrast-associated acute kidney injury; CKD-EPI, Chronic Kidney Disease Epidemiology Collaboration; eGFR, estimated glomerular filtration rate; HbA1c, glycated hemoglobin A1c; NSTE-ACS, non-ST-segment elevation acute coronary syndrome; SMD, standardized mean difference; STEMI, ST-segment elevation myocardial infarction.

### 3.2 Primary model performance and incremental value

After correlation and multicollinearity screening, four complementary feature-selection methods were applied in the Dali derivation cohort. Blood urea nitrogen, baseline serum creatinine, lymphocyte percentage, renin–angiotensin system inhibitor use, and uric acid were supported by all four methods, whereas age, fibrinogen, and glycated hemoglobin A1c were supported by three methods. These eight predictors were retained after clinical review and constituted the fixed predictor set used for model development and validation (Figure 2).

**Figure 3:**
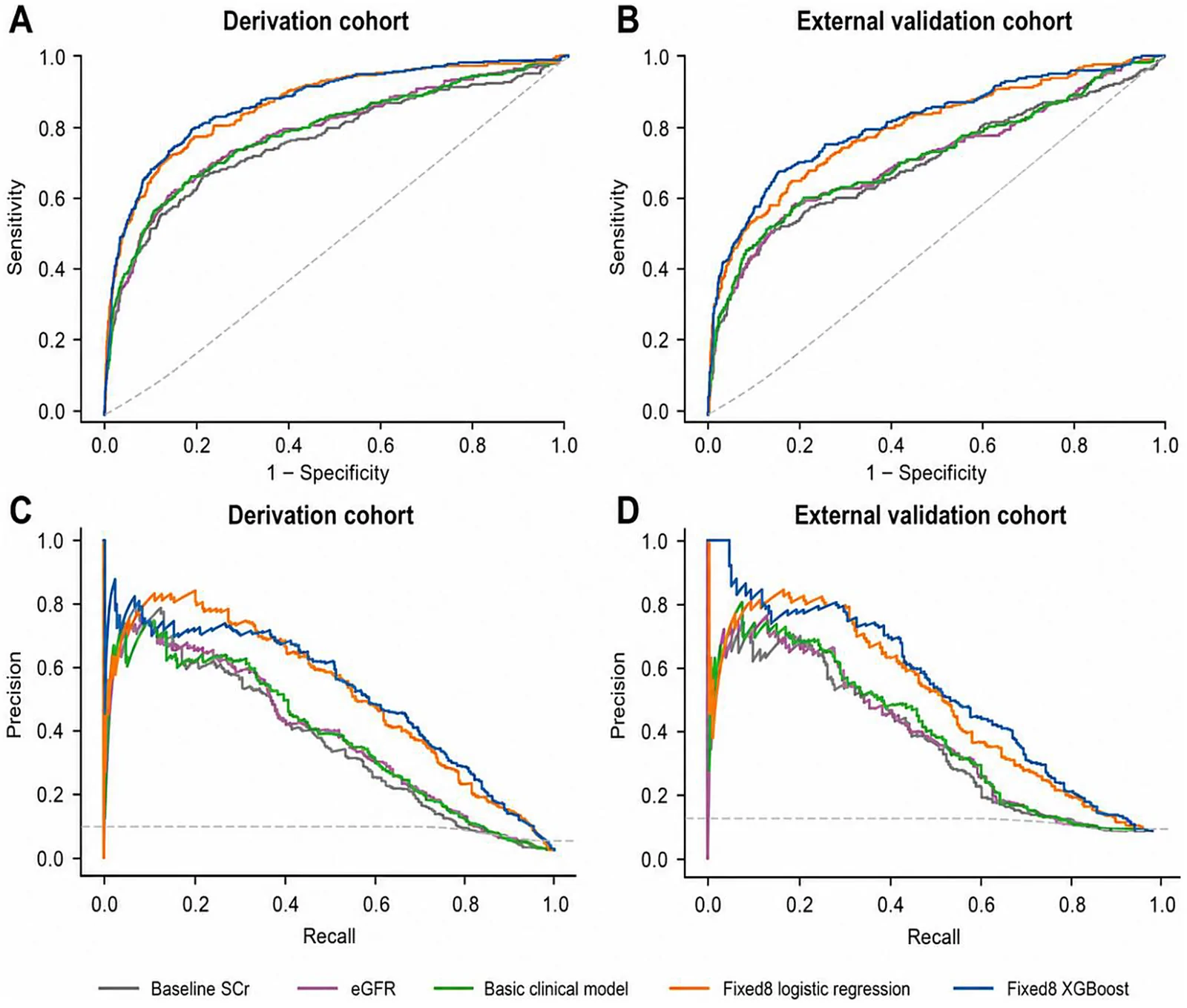
Discrimination of the clinical benchmark models and fixed eight-predictor models in the derivation and geographical external validation cohorts. Receiver operating characteristic curves are shown for the Dali derivation cohort using nested five-fold out-of-fold predictions after establishment of the predictor set **(A)** and the Baoshan geographical external validation cohort using predictions from the locked models without refitting **(B)** Precision–recall curves are shown for the corresponding derivation **(C)** and external validation **(D)** cohorts. The models included a baseline serum creatinine model, an estimated glomerular filtration rate model, a basic clinical model, fixed eight-predictor logistic regression, and fixed eight-predictor XGBoost. The basic clinical model included age, sex, diabetes mellitus, and estimated glomerular filtration rate. Diagonal reference lines in the receiver operating characteristic panels indicate no-discrimination performance, and horizontal reference lines in the precision–recall panels indicate the cohort-specific prevalence of CA-AKI. CA-AKI, contrast-associated acute kidney injury; eGFR, estimated glomerular filtration rate; SCr, serum creatinine; XGBoost, extreme gradient boosting.

After the predictor set had been established, nested five-fold cross-validation generated out-of-fold predictions in the Dali cohort. The fixed eight-predictor XGBoost model achieved an AUROC of 0.876 (95% CI, 0.853–0.897), an AUPRC of 0.565 (95% CI, 0.507–0.622), a Brier score of 0.067, and a scaled Brier score of 0.327. In the locked Baoshan external validation cohort, the corresponding values were 0.819 (95% CI, 0.786–0.851), 0.581 (95% CI, 0.522–0.637), 0.096, and 0.279, respectively (Table 2 and Figures 3A–D).

**Table 2:** Performance and incremental value of clinical benchmark and fixed eight-predictor models.

Panel A. Model performance							
Model	Internal OOF AUROC (95% CI)	External AUROC (95% CI)	External AUPRC (95% CI)	External Brier	External scaled Brier	External calibration slope	External O/E (95% CI)
Baseline SCr logistic	0.771 (0.740–0.804)	0.711 (0.670–0.751)	0.427 (0.372–0.494)	0.116	0.127	0.827	1.342 (1.179–1.522)
eGFR logistic	0.794 (0.762–0.824)	0.721 (0.680–0.759)	0.439 (0.382–0.505)	0.111	0.165	0.746	1.203 (1.056–1.364)
Basic clinical logistic	0.793 (0.761–0.823)	0.726 (0.683–0.765)	0.453 (0.396–0.520)	0.110	0.172	0.773	1.213 (1.065–1.375)
Fixed8 logistic	0.869 (0.844–0.891)	0.804 (0.769–0.834)	0.544 (0.487–0.611)	0.098	0.264	0.707	1.046 (0.919–1.186)
Fixed8 XGBoost	0.876 (0.853–0.897)	0.819 (0.786–0.851)	0.581 (0.522–0.637)	0.096	0.279	0.747	1.059 (0.930–1.201)

Panel B. Paired external-validation differences relative to Fixed8 XGBoost				
Comparator	*Δ*AUROC (95% CI)	*P* value	*Δ*AUPRC (95% CI)	*Δ*Brier score (95% CI)
Baseline SCr logistic	0.108 (0.076–0.142)	<0.001	0.155 (0.109–0.196)	−0.020 (−0.027–−0.014)
eGFR logistic	0.098 (0.069–0.129)	<0.001	0.142 (0.095–0.183)	−0.015 (−0.021–−0.009)
Basic clinical logistic	0.093 (0.063–0.123)	<0.001	0.128 (0.080–0.173)	−0.014 (−0.020–−0.008)
Fixed8 logistic	0.015 (0.002–0.030)	0.024	0.037 (0.005–0.065)	−0.002 (−0.005–0.001)

Internal performance was estimated from nested five-fold out-of-fold predictions after establishment of the predictor set in the Dali derivation cohort. External performance was evaluated once in the locked Baoshan cohort. The basic clinical model included age, sex, diabetes mellitus, and eGFR. Fixed8 models included age, blood urea nitrogen, baseline serum creatinine, fibrinogen, lymphocyte percentage, renin–angiotensin system inhibitor use, uric acid, and HbA1c. Scaled Brier score was calculated as 1−(model Brier score/null-model Brier score), with higher values indicating greater improvement over the prevalence-only model. In Panel B, differences are calculated as Fixed8 XGBoost minus the comparator; therefore, positive *Δ*AUROC and *Δ*AUPRC and negative *Δ*Brier favor Fixed8 XGBoost. Confidence intervals and *P* values were obtained using 2,000 paired bootstrap resamples. AUPRC, area under the precision–recall curve; AUROC, area under the receiver operating characteristic curve; CI, confidence interval; eGFR, estimated glomerular filtration rate; HbA1c, glycated hemoglobin A1c; O/E, observed-to-expected event ratio; OOF, out-of-fold; SCr, serum creatinine; XGBoost, extreme gradient boosting.

The fixed eight-predictor XGBoost model showed clear incremental discrimination over the three simpler clinical benchmarks in external validation. Relative to the baseline serum creatinine, eGFR, and basic clinical logistic models, the increases in AUROC were 0.108 (95% CI, 0.076–0.142), 0.098 (95% CI, 0.069–0.129), and 0.093 (95% CI, 0.063–0.123), respectively; all bootstrap *P* values were <0.001. The corresponding increases in AUPRC were 0.155, 0.142, and 0.128, and the Brier score was lower by 0.020, 0.015, and 0.014, respectively (Table 2).

The algorithmic increment over logistic regression using the same eight predictors was smaller. In internal validation, the XGBoost-minus-logistic difference in AUROC was 0.007 (95% CI, −0.005 to 0.018; *P* = 0.236), the difference in AUPRC was 0.005 (95% CI, −0.027 to 0.035), and the difference in Brier score was −0.001 (95% CI, −0.003 to 0.001). In external validation, the corresponding differences were 0.015 (95% CI, 0.002–0.030; *P* = 0.024), 0.037 (95% CI, 0.005–0.065), and −0.002 (95% CI, −0.005 to 0.001), respectively (Table 2).

### 3.3 Comparison across candidate algorithms and benchmark-score feasibility

The standardized comparison of 15 algorithms did not identify one algorithm that ranked first across all metrics and both cohorts. LightGBM had the highest internal AUROC (0.879), whereas the generalized additive model had the highest internal AUPRC (0.576) and the lowest internal Brier score (0.067). In external validation, AdaBoost had the highest AUROC (0.822), gradient boosting decision tree had the lowest Brier score (0.095), and XGBoost ranked third for AUROC, first for AUPRC, and second for Brier score. These rankings varied according to the selected metric and validation setting (Supplementary Table S3).

Reliable calculation of the original Mehran and Mehran 2 scores was not possible because essential variables were unavailable or not equivalent in definition, including contrast volume, hypotension, intra-aortic balloon pump use, and several procedural variables. No approximate scores were constructed (Supplementary Table S5).

### 3.4 Calibration and decision curve analysis

**Figure 4:**
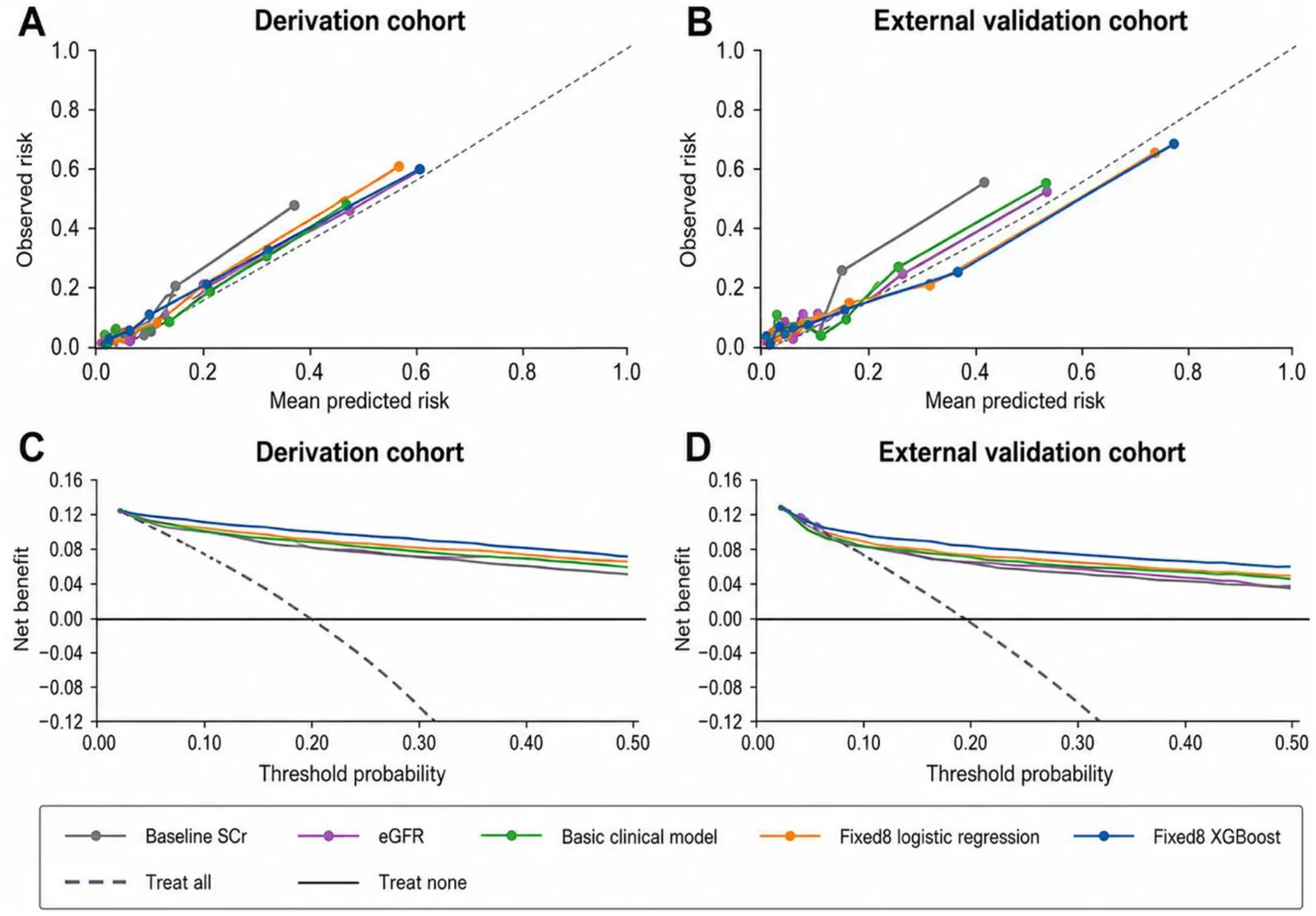
Calibration and clinical utility of the clinical benchmark models and fixed eight-predictor models. Calibration plots compare mean predicted and observed CA-AKI risks in the Dali derivation cohort using nested five-fold out-of-fold predictions after establishment of the predictor set **(A)** and in the Baoshan geographical external validation cohort using locked model predictions **(B)** The diagonal reference line represents perfect calibration. Decision curve analyses show net benefit across threshold probabilities from 0 to 0.50 in the derivation **(C)** and external validation **(D)** cohorts. The evaluated models were the baseline serum creatinine model, estimated glomerular filtration rate model, basic clinical model, fixed eight-predictor logistic regression, and fixed eight-predictor XGBoost. The treat-all and treat-none strategies are shown as clinical reference strategies. CA-AKI, contrast-associated acute kidney injury; DCA, decision curve analysis; eGFR, estimated glomerular filtration rate; SCr, serum creatinine; XGBoost, extreme gradient boosting.

The fixed eight-predictor XGBoost model showed close overall calibration in the derivation cohort, with a calibration-in-the-large of 0.014, a calibration slope of 1.034, and an O/E ratio of 1.009 (95% CI, 0.898–1.129). In the external validation cohort, calibration-in-the-large was 0.115, the calibration slope was 0.747, and the O/E ratio was 1.059 (95% CI, 0.930–1.201) (Table 2 and Figures 4A,B). The external calibration plot showed greater deviation from the ideal line at the lower predicted-risk levels than in the derivation cohort (Figure 4B).

Across threshold probabilities from 0.10 to 0.30, the fixed eight-predictor XGBoost model provided greater net benefit than the treat-all and treat-none strategies and the baseline serum creatinine, eGFR, and basic clinical models in both cohorts. The decision curves for fixed eight-predictor XGBoost, fixed eight-predictor logistic regression, and Routine4 XGBoost were closely aligned over much of this range (Figures 4C,D).

### 3.5 Exploratory model-based risk stratification

**Figure 5:**
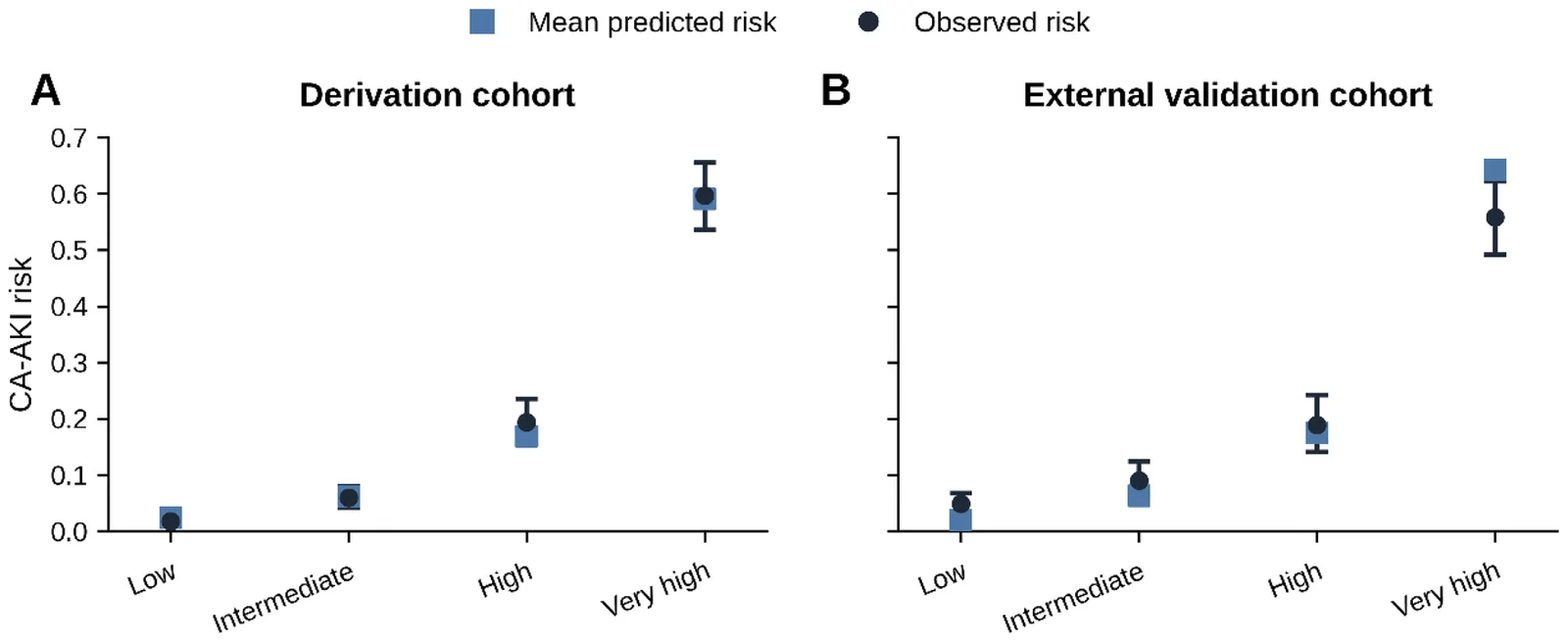
Observed and predicted CA-AKI risks across exploratory model-based risk categories. Mean predicted risks and observed CA-AKI risks are shown across the low-, intermediate-, high-, and very-high-risk categories in the Dali derivation cohort using nested five-fold out-of-fold predictions after establishment of the predictor set **(A)** and the Baoshan geographical external validation cohort **(B)**. Risk categories were defined using the 50th, 75th, and 90th percentiles of the out-of-fold predicted-risk distribution in the Dali cohort, and the resulting cutoffs were applied unchanged to the Baoshan cohort. Squares indicate mean predicted risk, circles indicate observed risk, and error bars represent 95% confidence intervals for observed risk. These categories were intended for exploratory model-based risk stratification and should not be interpreted as externally validated treatment thresholds. CA-AKI, contrast-associated acute kidney injury; CI, confidence interval.

Risk categories were defined using Dali out-of-fold probability cutoffs of 0.041, 0.099, and 0.311 and were applied unchanged to the Baoshan cohort. In Dali, observed CA-AKI risk increased from 1.8% (95% CI, 1.2%–2.7%) in the low-risk group to 6.0% (95% CI, 4.3%–8.1%), 19.4% (95% CI, 15.7%–23.6%), and 59.7% (95% CI, 53.6%–65.6%) in the intermediate-, high-, and very-high-risk groups, respectively. The corresponding observed risks in Baoshan were 4.9% (95% CI, 3.4%–6.8%), 9.0% (95% CI, 6.3%–12.5%), 18.9% (95% CI, 14.1%–24.3%), and 55.8% (95% CI, 49.2%–62.3%) (Table 3 and Figures 5A,B).

**Table 3:** Exploratory model-based risk stratification in the derivation and geographical external validation cohorts.

Cohort	Risk category	Patients, *n* (%)	CA-AKI events, n	Observed risk, % (95% CI)	Mean predicted risk, %	O/E ratio (95% CI)
Dali derivation cohort (OOF)	Low	1,338 (50.0)	24	1.8 (1.2–2.7)	2.4	0.759 (0.486–1.129)
	Intermediate	669 (25.0)	40	6.0 (4.3–8.1)	6.2	0.966 (0.690–1.315)
	High	402 (15.0)	78	19.4 (15.7–23.6)	16.9	1.148 (0.907–1.433)
	Very high	268 (10.0)	160	59.7 (53.6–65.6)	59.1	1.010 (0.860–1.179)
Baoshan external validation cohort	Low	697 (45.3)	34	4.9 (3.4–6.8)	2.0	2.426 (1.680–3.391)
	Intermediate	365 (23.7)	33	9.0 (6.3–12.5)	6.3	1.425 (0.981–2.001)
	High	244 (15.9)	46	18.9 (14.1–24.3)	17.5	1.080 (0.791–1.441)
	Very high	233 (15.1)	130	55.8 (49.2–62.3)	64.2	0.869 (0.726–1.032)

Risk categories were defined from the Dali Fixed8 XGBoost out-of-fold predicted probabilities using the 50th, 75th, and 90th percentiles and were fixed before application to the Baoshan cohort. The probability thresholds were 0.041, 0.099, and 0.311: low, <0.041; intermediate, 0.041 to <0.099; high, 0.099 to <0.311; and very high, ≥0.311. These categories are exploratory model-based risk categories and are not guideline-endorsed clinical thresholds. The external low- and intermediate-risk groups showed underprediction, most prominently in the low-risk group. CA-AKI, contrast-associated acute kidney injury; CI, confidence interval; O/E, observed-to-expected event ratio; OOF, out-of-fold; XGBoost, extreme gradient boosting.

Mean predicted risks in the four Baoshan categories were 2.0%, 6.3%, 17.5%, and 64.2%, respectively. The external O/E ratios were 2.426 (95% CI, 1.680–3.391) in the low-risk group, 1.425 (95% CI, 0.981–2.001) in the intermediate-risk group, 1.080 (95% CI, 0.791–1.441) in the high-risk group, and 0.869 (95% CI, 0.726–1.032) in the very-high-risk group (Table 3).

### 3.6 Missing data and sensitivity analyses

Among the fixed predictors, HbA1c had the greatest missingness: 44.3% in Dali and 82.6% in Baoshan. Fibrinogen was missing in 19.6% and 4.9% of patients, and lymphocyte percentage was missing in 6.1% and 3.8%, respectively. All eight predictors were observed in 1,186 Dali patients (44.3%) and 252 Baoshan patients (16.4%). CA-AKI occurred in 12.3% of Dali patients with HbA1c measured and 10.0% of those with HbA1c missing; the corresponding rates in Baoshan were 20.9% and 14.7% (Supplementary Tables S2, S7).

Reduced-predictor XGBoost models retained similar external performance. The model excluding HbA1c had an external AUROC of 0.821, AUPRC of 0.569, and Brier score of 0.095, and the model excluding HbA1c, fibrinogen, and uric acid had values of 0.821, 0.578, and 0.096, respectively. The Routine4 XGBoost model achieved an external AUROC of 0.832 (95% CI, 0.799–0.862), an AUPRC of 0.588 (95% CI, 0.527–0.649), and a Brier score of 0.094. Compared with Routine4 XGBoost, the fixed eight-predictor model had a lower external AUROC by 0.013 (95% CI, −0.024 to −0.002; *P* = 0.021); the difference in AUPRC was −0.006 (95% CI, −0.025 to 0.013), and the 95% CI for the Brier-score difference included zero (Supplementary Table S6).

In complete-case analyses, the fixed eight-predictor XGBoost model yielded an internal AUROC of 0.871 (95% CI, 0.836–0.905) and an external AUROC of 0.805 (95% CI, 0.732–0.875). In the external complete-case subset, the AUPRC was 0.607, the Brier score was 0.122, the calibration slope was 0.730, and the O/E ratio was 1.375 (95% CI, 1.021–1.813) (Supplementary Table S7).

### 3.7 Age-stratified performance

In the Baoshan cohort, 502 patients aged <60 years had 52 CA-AKI events (10.4%), whereas 1,036 patients aged ≥60 years had 191 events (18.4%); one participant with missing age was excluded from this analysis. The AUROC was 0.763 (95% CI, 0.680–0.835) among patients aged <60 years and 0.830 (95% CI, 0.794–0.865) among those aged ≥60 years. The corresponding AUPRC values were 0.415 and 0.625, and the O/E ratios were 1.387 (95% CI, 1.036–1.819) and 0.995 (95% CI, 0.859–1.147), respectively. The between-group AUROC difference for patients aged ≥60 years minus those aged <60 years was 0.067 (95% CI, −0.014 to 0.156; *P* = 0.111) (Supplementary Table S8 and Supplementary Figure S1).

### 3.8 Model interpretation

**Figure 6:**
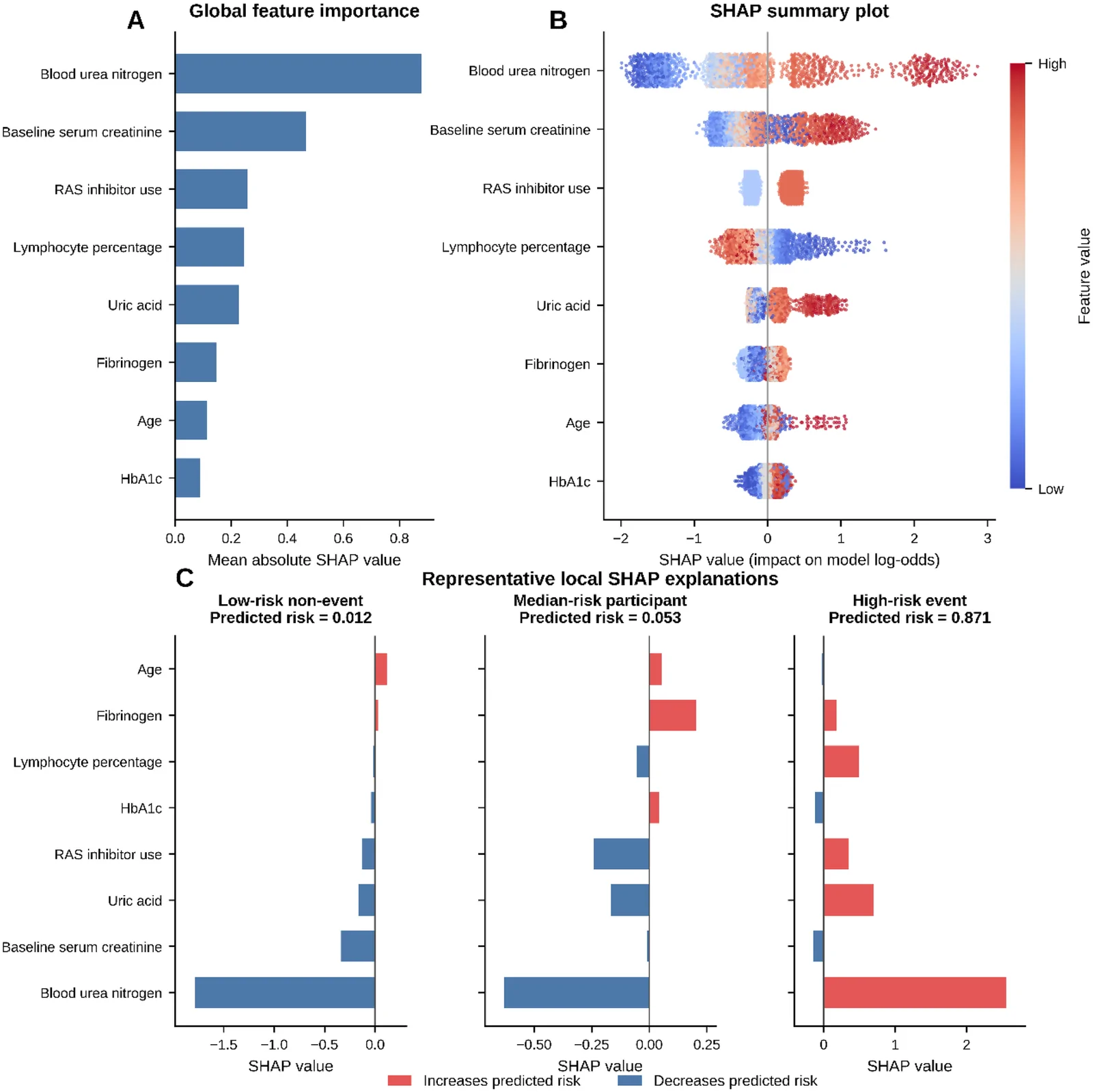
Global and individual interpretation of the fixed eight-predictor XGBoost model using SHAP values. **(A)** Global feature importance ranked according to the mean absolute SHAP value. **(B)** SHAP summary plot showing the direction and magnitude of each predictor's contribution to the model output in the derivation cohort. Each point represents one patient; positive SHAP values increase the model log-odds of CA-AKI, whereas negative values decrease the model log-odds. Red indicates higher feature values and blue indicates lower feature values. **(C)** Local SHAP explanations for three representative participants: a low-risk patient without CA-AKI, a participant with a predicted risk near the cohort median, and a high-risk patient who developed CA-AKI. Red bars indicate contributions that increase predicted risk, whereas blue bars indicate contributions that decrease predicted risk. The corresponding predicted risks were 0.012, 0.053, and 0.871, respectively. CA-AKI, contrast-associated acute kidney injury; SHAP, SHapley Additive exPlanations; XGBoost, extreme gradient boosting.

Global SHAP importance ranked blood urea nitrogen as the leading contributor to the fixed eight-predictor XGBoost model, followed by baseline serum creatinine, renin–angiotensin system inhibitor use, lymphocyte percentage, uric acid, fibrinogen, age, and HbA1c (Figure 6A). The SHAP summary and dependence plots showed that higher blood urea nitrogen and higher baseline serum creatinine generally contributed toward higher model output, whereas higher lymphocyte percentage generally contributed toward lower model output. Uric acid showed larger positive contributions at higher observed values (Figure 6B and Supplementary Figure S2).

The three representative local explanations had final predicted risks of 0.012 for a low-risk non-event, 0.053 for a participant near the median risk, and 0.871 for a high-risk event. In the high-risk event, blood urea nitrogen provided the largest positive contribution, with additional positive contributions from uric acid, lymphocyte percentage, and renin–angiotensin system inhibitor use (Figure 6C). Partial dependence plots showed increasing mean predicted risk at higher blood urea nitrogen and baseline serum creatinine values, decreasing predicted risk across higher lymphocyte percentages, and a rise in predicted risk at the upper range of uric acid. The remaining curves were comparatively flatter over most of their observed grids (Supplementary Figure S3).

## 4 Discussion

### 4.1 Principal findings

In this two-center retrospective prediction study, the fixed eight-predictor XGBoost model showed good discrimination during nested internal validation and retained useful discrimination when transferred without refitting to a geographically distinct hospital. The model improved substantially on serum creatinine, estimated glomerular filtration rate, and a basic clinical model, but its incremental performance over logistic regression using the same predictors was limited, indicating that much of the predictive information arose from the predictor set rather than from the nonlinear algorithm alone. Overall external calibration was reasonable, although the calibration slope and risk-category results indicated overfitting and underprediction in parts of the lower-risk range. Algorithm rankings varied by metric and cohort, and the parsimonious Routine4 model performed comparably in sensitivity analyses. Risk categories preserved a monotonic ordering across centers, whereas the numerical age-related performance difference remained statistically uncertain. SHAP and partial dependence analyses identified clinically plausible model-contribution patterns, but these findings describe model behavior rather than causal effects or validated clinical thresholds.

### 4.2 Comparison with previous studies

The limited advantage of XGBoost over same-variable logistic regression is consistent with broader evidence that machine learning does not automatically outperform conventional regression in structured clinical prediction problems (28). A meta-analysis focused on acute kidney injury similarly found that apparent machine-learning gains were sensitive to study quality, validation design, and the choice of comparator (29). These observations are relevant to the present study because only eight structured predictors were used, leaving less scope for complex algorithms to exploit high-dimensional interactions than in studies based on hundreds of electronic health record variables.

Previous machine-learning studies after percutaneous coronary intervention have reported encouraging discrimination, but their designs and information sets differ materially. Huang et al. used a large registry with extensive clinical and procedural information and showed that machine learning could improve post-PCI acute kidney injury prediction (30). Kuno et al. demonstrated that machine-learning models could preserve performance while using fewer variables from an established registry framework (31). More recent work has emphasized contemporary preprocedural risk estimation and the importance of evaluating models against strong clinical comparators rather than against weak baselines (32). A systematic review and meta-analysis of machine-learning models for kidney complications after coronary interventions also reported substantial heterogeneity in predictors, outcome definitions, validation methods, and performance estimates (33).

Studies conducted specifically in acute coronary syndrome populations have likewise produced variable results. Behnoush et al. reported acceptable performance across several algorithms, with feature rankings that depended on whether procedural and postprocedural information was available (34). Explainable deep-learning models have also been developed in patients with chronic kidney disease undergoing coronary angiography, but their different population, higher-dimensional architecture, and specialized validation setting limit direct comparison with the present model (35). In contrast, our analysis used a fixed low-dimensional predictor set, fold-specific preprocessing, nested out-of-fold predictions after establishment of the predictor set, a same-variable logistic comparator, and a fully locked cross-hospital evaluation. This more conservative framework may partly explain why the algorithmic increment was modest despite good overall discrimination.

The external findings also illustrate why discrimination alone is insufficient. Methodological guidance recommends that external validation assess calibration, prediction error, and clinical usefulness in addition to ranking ability (36). Calibration is especially vulnerable to differences in outcome prevalence, case mix, measurement practice, and care pathways across settings (22). Reviews of prediction models have shown that external performance is often worse than development performance (37), and this pattern is common in cardiovascular prediction research (38). In the present study, risk ordering remained clear in Baoshan, but the calibration slope was below one and the lower-risk categories were underpredicted. Therefore, the model demonstrated transportable ranking ability, not evidence that the original probabilities can be adopted unchanged for clinical deployment.

### 4.3 Potential mechanisms

The interpretation analyses suggested that renal-function-related variables accounted for much of the model signal. Higher blood urea nitrogen and baseline serum creatinine may represent reduced renal reserve, impaired perfusion, neurohormonal activation, catabolic stress, or a combination of these processes. In susceptible patients, iodinated contrast exposure may coincide with renal vasoconstriction, medullary hypoxia, and direct tubular stress (39). Oxidative stress and impaired antioxidant defenses could further amplify endothelial and tubular injury (40). Contemporary reviews emphasize that post-angiography acute kidney injury is usually multifactorial and may reflect hemodynamic instability, concurrent illness, and procedural factors in addition to contrast exposure itself (41). Thus, the model should not be interpreted as isolating a contrast-specific biological pathway.

Age may capture declining nephron reserve, vascular stiffness, frailty, and a higher burden of comorbidity, although the present age-stratified analysis did not establish statistically significant heterogeneity. Prior pooled data in older patients with acute coronary syndrome indicate that CA-AKI remains clinically consequential in this population (42). The numerical difference between age groups in our study may therefore reflect differences in event prevalence, case mix, or calibration as much as true age-dependent model behavior.

Uric acid showed increasingly positive SHAP contributions at higher values. Hyperuricemia has been associated with contrast-related acute kidney injury in meta-analysis and may plausibly reflect impaired renal excretion, oxidative stress, endothelial dysfunction, and intrarenal vasoconstriction (43). However, serum uric acid is also strongly influenced by baseline kidney function, diuretic exposure, metabolic disease, and acute illness; its model importance cannot establish an independent or modifiable causal effect.

The inverse contribution pattern for lymphocyte percentage may reflect systemic stress and inflammatory activation rather than a protective effect of lymphocytes. A meta-analysis of hematological and inflammatory indices reported associations between inflammatory profiles and contrast-related kidney injury after coronary intervention (44). Composite immune-inflammatory indices have also shown predictive value in acute coronary syndrome cohorts undergoing intervention (45). Because lymphocyte percentage is affected by infection, corticosteroid exposure, physiological stress, and changes in other leukocyte fractions, its SHAP pattern should be viewed as a marker of the model's inflammatory information rather than a mechanism proven by this study.

Fibrinogen may integrate acute-phase activity, blood viscosity, endothelial dysfunction, and prothrombotic burden. Elevated fibrinogen has previously been associated with contrast-related acute kidney injury in patients treated invasively for acute coronary syndrome (46). HbA1c may reflect chronic dysglycemia, microvascular injury, and reduced renal resilience, whereas acute hyperglycemia has also been associated with higher contrast-related kidney injury risk (47). Diabetes itself is a recognized susceptibility factor across large contrast-exposed populations (48). Blood urea nitrogen may additionally summarize systemic vulnerability beyond glomerular filtration, and BUN-based indices have been associated with contrast-related nephropathy in coronary disease cohorts (49). Nevertheless, umbrella evidence shows that biomarker associations vary by population, assay timing, adjustment strategy, and endpoint definition (50). The comparatively small contribution of renin-angiotensin system inhibitor use should likewise be interpreted as a predictive association that may encode treatment indication and case mix, not as evidence for benefit or harm from the medication.

### 4.4 Clinical and research implications

The model may help identify patients who warrant closer renal assessment and postprocedural creatinine surveillance, but it should not be used to delay indicated coronary angiography or to trigger unvalidated treatment thresholds. Its main contribution is the preservation of risk ranking across hospitals using a compact predictor set. Recent work in NSTEMI triage illustrates how externally evaluated machine-learning probabilities can be translated into rule-out and rule-in strata and integrated with established clinical pathways (51). After prospective validation, calibrated risk estimates could support clinician–patient or family discussions regarding renal risk and postprocedural monitoring. The present study did not evaluate shared decision-making or patient-facing risk communication. Nevertheless, the limited gain over same-variable logistic regression, closely overlapping decision curves, and the higher external AUROC of the Routine4 model argue against assuming that the full XGBoost model is the preferred implementation strategy. Future studies should prospectively compare the full and parsimonious models, evaluate local recalibration, standardize measurement timing and procedural variables, and test whether model-assisted workflows improve clinical decisions or patient outcomes. When the model is evaluated in settings with different laboratory practices or event prevalence, prediction uncertainty and predictor availability should be reported.

### 4.5 Strengths and limitations

Strengths included nested internal validation, a locked geographical external cohort, clinically relevant comparators, comprehensive calibration assessment, and complementary model-interpretation and sensitivity analyses.

Several limitations remain. First, the retrospective design and requirement for available creatinine fields may have introduced selection bias and limited representation of patients with incomplete monitoring. Because feature selection was performed using the complete Dali derivation cohort before nested model validation, the internal performance estimates may retain some selection-related optimism; the locked Baoshan evaluation therefore provides the more conservative assessment of transportability. Second, although CA-AKI was ascertained using the baseline serum creatinine measurement before coronary angiography and the follow-up serum creatinine measurement obtained 48–72 h after the procedure, the retrospective nature of the data may still have introduced variability in the timing and frequency of creatinine testing. Accordingly, some measurement error or outcome underascertainment remains possible. Furthermore, CA-AKI denotes acute kidney injury occurring after contrast exposure and does not, by itself, establish a direct causal effect of contrast media. Third, important procedural and preventive variables, including contrast volume, hemodynamic instability, hydration strategy, and intra-aortic balloon pump use, were unavailable or not comparable, precluding reliable reconstruction of the Mehran scores and potentially omitting relevant predictive information. Fourth, HbA1c was frequently missing, particularly in the external cohort, and median imputation assumes that the observed predictor distribution provides an adequate substitute for unavailable values. Methods for handling missing predictors can materially affect transportability and should be evaluated in the intended deployment workflow (52). The complete-case subset was small and cannot resolve informative missingness. Fifth, both hospitals were located in Yunnan Province, and no independently verifiable temporal or prospective validation was available. The external calibration findings indicate that local recalibration may be required elsewhere. The model was restricted to structured EHR variables and did not incorporate unstructured symptoms or physical-examination findings. Future studies could evaluate privacy-preserving, cross-center natural-language processing pipelines before incorporating such information into prediction workflows. Finally, the age analysis was targeted and imprecise, and the absence of a statistically significant difference does not establish equivalence across age groups. SHAP and partial dependence plots remain vulnerable to correlated predictors, confounding, and model-specific artifacts and should not be interpreted causally.

## 5 Conclusions

A fixed eight-predictor XGBoost model provided good internal discrimination and retained useful risk ranking in a locked geographical external cohort of patients with acute coronary syndrome undergoing coronary angiography. It improved clearly on simple clinical benchmarks, but its advantage over same-variable logistic regression was limited, and external calibration was imperfect. Routine4 achieved a slightly higher external AUROC; substantial predictor missingness further tempers claims of immediate implementation. Prospective multicenter validation, standardized measurement timing, local calibration assessment, and clinical-impact evaluation are required before routine use.

## Data Availability

The datasets analyzed during the current study are not publicly available due to institutional privacy policies and patient confidentiality restrictions but are available from the corresponding author upon reasonable request and with permission from the participating hospitals.
