# Supplementary material for: An interpretable XGBoost model for contrast-associated acute kidney injury in patients with acute coronary syndrome: development and geographical external validation
Source: Front Digit Health. 2026 Aug 18;8:1917647. doi: 10.3389/fdgth.2026.1917647 (PMC13529729; doi:10.3389/fdgth.2026.1917647)
Supplement: Supplementary file 1 [file Table1.docx]

| **Supplementary Table S1. Definitions, availability, and preprocessing of the fixed eight predictors** | | | | | | |
| --- | --- | --- | --- | --- | --- | --- |
| **Predictor** | **Definition** | **Unit/category** | **Model coding** | **Dali missing** | **Baoshan missing** | **Preprocessing** |
| Age | Age at the index admission. | years | Continuous | 0 (0.0%) | 1 (0.1%) | Training-fold median imputation; final full-Dali median = 60 |
| Blood urea nitrogen | Available blood urea nitrogen value in the harmonized analytical dataset. | mmol/L | Continuous | 0 (0.0%) | 0 (0.0%) | Training-fold median imputation; final full-Dali median = 5.79 |
| Baseline serum creatinine | Available serum creatinine value designated as baseline in the harmonized analytical dataset. | μmol/L | Continuous | 0 (0.0%) | 0 (0.0%) | Training-fold median imputation; final full-Dali median = 77 |
| Fibrinogen | Available plasma fibrinogen value in the harmonized analytical dataset. | g/L | Continuous | 524 (19.6%) | 75 (4.9%) | Training-fold median imputation; final full-Dali median = 3.27 |
| Lymphocyte percentage | Available peripheral-blood lymphocyte percentage in the harmonized analytical dataset. | % | Continuous | 164 (6.1%) | 58 (3.8%) | Training-fold median imputation; final full-Dali median = 22.5 |
| RAS inhibitor use | Recorded use of an ACE inhibitor, angiotensin receptor blocker, or angiotensin receptor–neprilysin inhibitor in the harmonized analytical dataset. | No/Yes | Binary 0/1 | 0 (0.0%) | 0 (0.0%) | Training-fold median imputation; final full-Dali median = 0 |
| Uric acid | Available serum uric acid value in the harmonized analytical dataset. | μmol/L | Continuous | 0 (0.0%) | 0 (0.0%) | Training-fold median imputation; final full-Dali median = 359 |
| Glycated hemoglobin A1c | Available glycated hemoglobin A1c value in the harmonized analytical dataset. | % | Continuous | 1185 (44.3%) | 1271 (82.6%) | Training-fold median imputation; final full-Dali median = 6.625 |
| **Note:** The eight-predictor set was established in the complete Dali derivation cohort before nested model validation and was subsequently used unchanged in all internal and geographical external validation analyses. Median imputation was estimated separately within each training fold during internal validation. The displayed full-Dali medians were used only when fitting the final locked model after model development was completed and were then applied unchanged to the Baoshan cohort; no imputation parameter was estimated from the external cohort. Except for the serum creatinine measurements used to ascertain CA-AKI, the exact timing of other laboratory and medication predictors relative to coronary angiography could not be consistently verified across the two hospital information systems. ACE, angiotensin-converting enzyme; CA-AKI, contrast-associated acute kidney injury; RAS, renin–angiotensin system. | | | | | | |

| **Supplementary Table S2. Candidate-predictor missingness and patient-level missing-data burden** | | | | | | |
| --- | --- | --- | --- | --- | --- | --- |
| **Panel A. Variable-level missingness** | | | | | | |
| **Candidate predictor** | | **Fixed8 predictor** | **Dali missing** | **Baoshan missing** | | |
| Age | | Yes | 0 (0.0%) | 1 (0.1%) | | |
| Sex | | No | 0 (0.0%) | 1 (0.1%) | | |
| Body mass index | | No | 0 (0.0%) | 362 (23.5%) | | |
| Smoking status | | No | 0 (0.0%) | 0 (0.0%) | | |
| Alcohol use | | No | 0 (0.0%) | 0 (0.0%) | | |
| ST-segment elevation myocardial infarction | | No | 0 (0.0%) | 0 (0.0%) | | |
| Killip class | | No | 0 (0.0%) | 898 (58.3%) | | |
| Hypertension | | No | 0 (0.0%) | 0 (0.0%) | | |
| Diabetes mellitus | | No | 0 (0.0%) | 0 (0.0%) | | |
| Heart failure | | No | 0 (0.0%) | 0 (0.0%) | | |
| Atrial fibrillation | | No | 0 (0.0%) | 0 (0.0%) | | |
| Cerebral infarction | | No | 0 (0.0%) | 0 (0.0%) | | |
| Peripheral artery disease | | No | 0 (0.0%) | 0 (0.0%) | | |
| Anemia | | No | 0 (0.0%) | 0 (0.0%) | | |
| Infection | | No | 0 (0.0%) | 0 (0.0%) | | |
| Cancer | | No | 0 (0.0%) | 0 (0.0%) | | |
| Chronic lung disease | | No | 0 (0.0%) | 0 (0.0%) | | |
| Respiratory failure | | No | 0 (0.0%) | 0 (0.0%) | | |
| Baseline serum creatinine | | Yes | 0 (0.0%) | 0 (0.0%) | | |
| Estimated glomerular filtration rate | | No | 0 (0.0%) | 1 (0.1%) | | |
| Blood urea nitrogen | | Yes | 0 (0.0%) | 0 (0.0%) | | |
| Uric acid | | Yes | 0 (0.0%) | 0 (0.0%) | | |
| Glucose | | No | 71 (2.7%) | 919 (59.7%) | | |
| Glycated hemoglobin A1c | | Yes | 1185 (44.3%) | 1271 (82.6%) | | |
| Total cholesterol | | No | 56 (2.1%) | 445 (28.9%) | | |
| Triglycerides | | No | 56 (2.1%) | 446 (29.0%) | | |
| High-density lipoprotein cholesterol | | No | 56 (2.1%) | 445 (28.9%) | | |
| Low-density lipoprotein cholesterol | | No | 56 (2.1%) | 445 (28.9%) | | |
| Albumin | | No | 31 (1.2%) | 320 (20.8%) | | |
| White blood cell count | | No | 164 (6.1%) | 58 (3.8%) | | |
| Neutrophil count | | No | 164 (6.1%) | 58 (3.8%) | | |
| Lymphocyte count | | No | 164 (6.1%) | 58 (3.8%) | | |
| Hemoglobin | | No | 164 (6.1%) | 58 (3.8%) | | |
| Platelet count | | No | 164 (6.1%) | 58 (3.8%) | | |
| Mean corpuscular volume | | No | 164 (6.1%) | 58 (3.8%) | | |
| Neutrophil percentage | | No | 164 (6.1%) | 58 (3.8%) | | |
| Lymphocyte percentage | | Yes | 164 (6.1%) | 58 (3.8%) | | |
| Monocyte percentage | | No | 164 (6.1%) | 58 (3.8%) | | |
| Hematocrit | | No | 164 (6.1%) | 222 (14.4%) | | |
| Red cell distribution width–coefficient of variation | | No | 164 (6.1%) | 58 (3.8%) | | |
| Potassium | | No | 20 (0.7%) | 57 (3.7%) | | |
| Sodium | | No | 20 (0.7%) | 57 (3.7%) | | |
| Chloride | | No | 20 (0.7%) | 57 (3.7%) | | |
| Calcium | | No | 20 (0.7%) | 57 (3.7%) | | |
| N-terminal pro-B-type natriuretic peptide | | No | 1849 (69.1%) | 910 (59.1%) | | |
| Alanine aminotransferase | | No | 31 (1.2%) | 320 (20.8%) | | |
| Aspartate aminotransferase | | No | 31 (1.2%) | 187 (12.2%) | | |
| Total bilirubin | | No | 31 (1.2%) | 320 (20.8%) | | |
| D-dimer | | No | 833 (31.1%) | 779 (50.6%) | | |
| Fibrinogen | | Yes | 524 (19.6%) | 75 (4.9%) | | |
| Prothrombin time | | No | 522 (19.5%) | 75 (4.9%) | | |
| Activated partial thromboplastin time | | No | 524 (19.6%) | 75 (4.9%) | | |
| International normalized ratio | | No | 522 (19.5%) | 75 (4.9%) | | |
| Left ventricular ejection fraction | | No | 163 (6.1%) | 317 (20.6%) | | |
| Left ventricular end-diastolic diameter | | No | 0 (0.0%) | 317 (20.6%) | | |
| Left atrial enlargement | | No | 163 (6.1%) | 317 (20.6%) | | |
| Left ventricular enlargement | | No | 163 (6.1%) | 317 (20.6%) | | |
| Pulmonary hypertension | | No | 0 (0.0%) | 317 (20.6%) | | |
| Antiplatelet strategy | | No | 0 (0.0%) | 0 (0.0%) | | |
| RAS inhibitor use | | Yes | 0 (0.0%) | 0 (0.0%) | | |
| β-blocker use | | No | 0 (0.0%) | 0 (0.0%) | | |
| Dihydropyridine calcium-channel blocker use | | No | 0 (0.0%) | 0 (0.0%) | | |
| Diuretic use | | No | 0 (0.0%) | 0 (0.0%) | | |
| Sodium–glucose cotransporter 2 inhibitor use | | No | 0 (0.0%) | 0 (0.0%) | | |
| Metformin use | | No | 0 (0.0%) | 0 (0.0%) | | |
| Thiazolidinedione use | | No | 0 (0.0%) | 0 (0.0%) | | |
| α-glucosidase inhibitor use | | No | 0 (0.0%) | 0 (0.0%) | | |
| Insulin use | | No | 0 (0.0%) | 0 (0.0%) | | |
| Percutaneous coronary intervention | | No | 0 (0.0%) | 685 (44.5%) | | |
| **Panel B. Patient-level burden for the fixed eight predictors** | | | | | | |
| **Cohort** | **n** | **Complete Fixed8** | **Missing ≥1** | **Missing ≥2** | **Maximum missing predictors** |  |
| Dali | 2677 | 1186 (44.3%) | 1491 (55.7%) | 322 (12.0%) | 3 |  |
| Baoshan | 1539 | 252 (16.4%) | 1287 (83.6%) | 107 (7.0%) | 3 |  |
| **Note:** Missingness was evaluated in the locked, un-imputed cohorts. Fixed8 denotes the fixed eight-predictor model. Outcome-defining and post-procedural serum creatinine variables were not included as candidate predictors. | | | | | | |

| **Supplementary Table S3. Performance of the 15 candidate algorithms under the standardized validation framework** | | | | | | | | |
| --- | --- | --- | --- | --- | --- | --- | --- | --- |
| **Model** | **Internal AUROC (95% CI)** | **Internal AUPRC (95% CI)** | **Internal Brier** | **Internal scaled Brier** | **External AUROC (95% CI)** | **External AUPRC (95% CI)** | **External Brier** | **External scaled Brier** |
| XGBoost | 0.876 (0.853–0.897) | 0.565 (0.507–0.622) | 0.067 | 0.327 | 0.819 (0.786–0.851) | 0.581 (0.522–0.637) | 0.096 | 0.279 |
| Generalized additive model | 0.874 (0.851–0.896) | 0.576 (0.522–0.635) | 0.067 | 0.330 | 0.812 (0.779–0.844) | 0.561 (0.496–0.627) | 0.097 | 0.271 |
| Gaussian process classifier | 0.843 (0.817–0.869) | 0.478 (0.430–0.541) | 0.084 | 0.160 | 0.789 (0.755–0.823) | 0.482 (0.426–0.550) | 0.110 | 0.173 |
| Gradient boosting decision tree | 0.877 (0.853–0.898) | 0.571 (0.518–0.629) | 0.068 | 0.324 | 0.820 (0.788–0.852) | 0.569 (0.512–0.634) | 0.095 | 0.282 |
| Random forest | 0.873 (0.849–0.894) | 0.566 (0.514–0.626) | 0.079 | 0.207 | 0.811 (0.776–0.842) | 0.539 (0.480–0.613) | 0.114 | 0.141 |
| Support vector machine | 0.865 (0.840–0.887) | 0.490 (0.442–0.550) | 0.072 | 0.279 | 0.785 (0.749–0.819) | 0.440 (0.388–0.505) | 0.109 | 0.180 |
| Naive Bayes | 0.858 (0.832–0.881) | 0.535 (0.482–0.593) | 0.085 | 0.155 | 0.788 (0.747–0.824) | 0.543 (0.485–0.610) | 0.108 | 0.189 |
| LightGBM | 0.879 (0.856–0.901) | 0.552 (0.497–0.613) | 0.068 | 0.323 | 0.814 (0.779–0.845) | 0.567 (0.506–0.633) | 0.096 | 0.278 |
| Rotation forest | 0.865 (0.841–0.889) | 0.552 (0.499–0.616) | 0.076 | 0.237 | 0.810 (0.774–0.841) | 0.554 (0.495–0.618) | 0.142 | -0.065 |
| Logistic regression | 0.869 (0.844–0.891) | 0.560 (0.506–0.619) | 0.069 | 0.315 | 0.804 (0.769–0.834) | 0.544 (0.487–0.611) | 0.098 | 0.264 |
| AdaBoost | 0.832 (0.804–0.860) | 0.522 (0.469–0.585) | 0.079 | 0.214 | 0.822 (0.789–0.854) | 0.542 (0.485–0.604) | 0.100 | 0.244 |
| Extremely randomized trees | 0.870 (0.848–0.891) | 0.549 (0.495–0.606) | 0.144 | -0.439 | 0.774 (0.738–0.810) | 0.512 (0.447–0.577) | 0.185 | -0.393 |
| Multilayer perceptron | 0.839 (0.812–0.863) | 0.527 (0.474–0.591) | 0.074 | 0.258 | 0.782 (0.747–0.815) | 0.499 (0.443–0.572) | 0.106 | 0.202 |
| k-nearest neighbors | 0.840 (0.812–0.865) | 0.534 (0.478–0.593) | 0.073 | 0.272 | 0.790 (0.757–0.826) | 0.541 (0.481–0.607) | 0.100 | 0.251 |
| Decision tree | 0.827 (0.798–0.855) | 0.485 (0.430–0.542) | 0.144 | -0.437 | 0.807 (0.771–0.839) | 0.513 (0.457–0.574) | 0.160 | -0.205 |
| **Note:** Internal performance was estimated from nested five-fold out-of-fold predictions after establishment of the predictor set in the Dali derivation cohort. The Baoshan cohort was used only for locked geographical external validation. Scaled Brier score = 1 − model Brier score/null-model Brier score. AUROC, area under the receiver operating characteristic curve; AUPRC, area under the precision–recall curve; CI, confidence interval. | | | | | | | | |

| **Supplementary Table S4. Hyperparameter optimization, model implementation, and software environment** | | | | |
| --- | --- | --- | --- | --- |
| **Panel A. Common validation and computing framework** | | | | |
| **Component** | | **Specification** | | |
| Fixed predictor set | | Age, blood urea nitrogen, baseline serum creatinine, fibrinogen, lymphocyte percentage, RAS inhibitor use, uric acid, and HbA1c | | |
| Outer validation | | Stratified five-fold cross-validation with shuffling; random seed 20260715 | | |
| Inner tuning | | Stratified three-fold cross-validation within each outer-training fold | | |
| Optimization objective | | Negative log loss | | |
| Preprocessing | | Median imputation fitted within the training fold; standardization within fold for algorithms requiring scaling | | |
| External validation | | Baoshan predictions generated without refitting, recalibration, threshold selection, or model selection | | |
| Bootstrap inference | | 2000 stratified paired bootstrap replicates | | |
| Software environment | | Python 3.11.15; numpy 1.26.4; pandas 2.3.3; scipy 1.16.3; scikit-learn 1.8.0; statsmodels 0.14.6; xgboost 3.2.0; lightgbm 4.6.0; pygam 0.12.0 | | |
| **Panel B. Model-specific implementation and tuning** | | | | |
| **Model** | **Implementation** | **Preprocessing** | **Search space** | **Final hyperparameters** |
| XGBoost | xgboost XGBClassifier | Median imputation within training fold | n_estimators: 100, 200; max_depth: 2, 3; learning_rate: 0.03, 0.05; min_child_weight: 1, 5 | learning_rate=0.03; max_depth=3; min_child_weight=1; n_estimators=200 |
| Generalized additive model | pygam-based generalized additive classifier | Median imputation within training fold; standardization within fold | lam: 1, 10; n_splines: 5, 10 | lam=1; n_splines=10 |
| Gaussian process classifier | scikit-learn GaussianProcessClassifier with fixed RBF kernel | Median imputation within training fold; standardization within fold | None | None |
| Gradient boosting decision tree | scikit-learn GradientBoostingClassifier | Median imputation within training fold | n_estimators: 100, 200; max_depth: 2, 3; learning_rate: 0.03, 0.05 | learning_rate=0.05; max_depth=2; n_estimators=100 |
| Random forest | scikit-learn RandomForestClassifier | Median imputation within training fold | max_depth: 5, None; min_samples_leaf: 5, 20 | max_depth=None; min_samples_leaf=5 |
| Support vector machine | scikit-learn support vector classifier | Median imputation within training fold; standardization within fold | C: 0.1, 1; gamma: scale, 0.1 | C=0.1; gamma=0.1 |
| Naive Bayes | scikit-learn GaussianNB | Median imputation within training fold | var_smoothing: 1e-11, 1e-09, 1e-07 | var_smoothing=1e-07 |
| LightGBM | lightgbm LGBMClassifier | Median imputation within training fold | n_estimators: 100, 200; max_depth: 3, 5; learning_rate: 0.03, 0.05; num_leaves: 7, 15 | learning_rate=0.05; max_depth=5; n_estimators=100; num_leaves=7 |
| Rotation forest | Custom canonical-style rotation forest | Median imputation within training fold; standardization within fold | n_estimators: 30, 60; group_size: 2, 3; max_depth: 5, None; min_samples_leaf: 5, 20 | group_size=2; max_depth=None; min_samples_leaf=20; n_estimators=60 |
| Logistic regression | scikit-learn LogisticRegression | Median imputation within training fold; standardization within fold | C: 0.1, 1, 10 | C=1.0 |
| AdaBoost | scikit-learn AdaBoostClassifier | Median imputation within training fold | n_estimators: 50, 100; learning_rate: 0.03, 0.1 | learning_rate=0.03; n_estimators=50 |
| Extremely randomized trees | scikit-learn ExtraTreesClassifier | Median imputation within training fold | max_depth: 5, None; min_samples_leaf: 5, 20 | max_depth=None; min_samples_leaf=5 |
| Multilayer perceptron | scikit-learn MLPClassifier | Median imputation within training fold; standardization within fold | hidden_layer_sizes: [32], [50, 20]; alpha: 0.0001, 0.01 | alpha=0.01; hidden_layer_sizes: 50, 20 |
| k-nearest neighbors | scikit-learn KNeighborsClassifier | Median imputation within training fold; standardization within fold | n_neighbors: 7, 15, 31; weights: uniform, distance | n_neighbors=31; weights=distance |
| Decision tree | scikit-learn DecisionTreeClassifier | Median imputation within training fold | max_depth: 3, 5, None; min_samples_leaf: 10, 30 | max_depth=3; min_samples_leaf=10 |
| **Note:** All search spaces were fixed before the locked external evaluation. RBF, radial basis function. | | | | |

| **Supplementary Table S5. Feasibility assessment of the original Mehran and Mehran 2 risk scores** | | | | |
| --- | --- | --- | --- | --- |
| **Risk score** | **Required item** | **Availability in current data** | **Reason/limitation** | **Reliable calculation** |
| Original Mehran | Contrast volume | absent | Cannot calculate | No |
| Original Mehran | Hypotension | absent | Cannot calculate | No |
| Original Mehran | IABP | absent | Cannot calculate | No |
| Original Mehran | Congestive heart failure/pulmonary edema | Available field not equivalent in definition/timing | Cannot calculate | No |
| Original Mehran | Age >75 | available | Insufficient without other items | No |
| Original Mehran | Anemia | Hematocrit and sex available | Reliable reconstruction remained impossible because other mandatory score components were absent | No |
| Original Mehran | Diabetes | available | Insufficient | No |
| Original Mehran | Renal dysfunction | eGFR derivable with 2021 equation | Not original-era equation | No |
| Mehran 2 | Detailed ACS subtype | STEMI versus combined NSTE-ACS available | NSTEMI could not be distinguished from unstable angina | No |
| Mehran 2 | Procedural variables | absent | Contrast volume/bleeding/slow flow/complex anatomy unavailable | No |
| **Note:** Approximate scores were not constructed because doing so would require unvalidated substitutions or assumptions. The study therefore compared the fixed eight-predictor model with baseline serum creatinine, eGFR, a basic clinical model, and logistic regression using the same eight predictors. IABP, intra-aortic balloon pump; NSTE-ACS, non-ST-segment elevation acute coronary syndrome; NSTEMI, non-ST-segment elevation myocardial infarction; STEMI, ST-segment elevation myocardial infarction. | | | | |

| **Supplementary Table S6. Reduced-model and predictor-availability sensitivity analyses** | | | | | | | | | | | | |
| --- | --- | --- | --- | --- | --- | --- | --- | --- | --- | --- | --- | --- |
| **Panel A. Reduced-model performance** | | | | | | | | | | | | |
| **Model** | **Cohort** | | **n/events** | **AUROC (95% CI)** | | **AUPRC (95% CI)** | **Brier** | **Scaled Brier** | **Calibration slope** | | **O/E (95% CI)** |  |
| No-HbA1c logistic regression | Dali OOF | | 2677/302 | 0.869 (0.845–0.891) | | 0.560 (0.504–0.622) | 0.068 | 0.316 | 0.988 | | 1.000 (0.890–1.119) |  |
| No-HbA1c XGBoost | Dali OOF | | 2677/302 | 0.878 (0.857–0.900) | | 0.552 (0.499–0.615) | 0.068 | 0.324 | 0.982 | | 1.005 (0.895–1.125) |  |
| No-HbA1c/fibrinogen/uric acid logistic regression | Dali OOF | | 2677/302 | 0.864 (0.839–0.887) | | 0.550 (0.496–0.613) | 0.070 | 0.304 | 0.998 | | 1.000 (0.891–1.120) |  |
| No-HbA1c/fibrinogen/uric acid XGBoost | Dali OOF | | 2677/302 | 0.872 (0.849–0.893) | | 0.555 (0.498–0.616) | 0.068 | 0.321 | 0.983 | | 1.007 (0.897–1.127) |  |
| Routine4 logistic regression | Dali OOF | | 2677/302 | 0.852 (0.827–0.876) | | 0.546 (0.491–0.608) | 0.070 | 0.299 | 0.975 | | 1.001 (0.891–1.121) |  |
| Routine4 XGBoost | Dali OOF | | 2677/302 | 0.865 (0.842–0.888) | | 0.553 (0.493–0.613) | 0.069 | 0.313 | 0.993 | | 1.005 (0.895–1.125) |  |
| No-HbA1c logistic regression | Baoshan external | | 1539/243 | 0.804 (0.770–0.835) | | 0.545 (0.486–0.611) | 0.098 | 0.265 | 0.709 | | 1.037 (0.911–1.176) |  |
| No-HbA1c XGBoost | Baoshan external | | 1539/243 | 0.821 (0.788–0.852) | | 0.569 (0.508–0.633) | 0.095 | 0.284 | 0.780 | | 1.056 (0.927–1.197) |  |
| No-HbA1c/fibrinogen/uric acid logistic regression | Baoshan external | | 1539/243 | 0.810 (0.775–0.842) | | 0.546 (0.486–0.615) | 0.097 | 0.269 | 0.774 | | 1.030 (0.905–1.168) |  |
| No-HbA1c/fibrinogen/uric acid XGBoost | Baoshan external | | 1539/243 | 0.821 (0.786–0.851) | | 0.578 (0.519–0.638) | 0.096 | 0.277 | 0.736 | | 1.039 (0.912–1.178) |  |
| Routine4 logistic regression | Baoshan external | | 1539/243 | 0.822 (0.788–0.855) | | 0.560 (0.500–0.625) | 0.095 | 0.283 | 0.814 | | 1.068 (0.938–1.211) |  |
| Routine4 XGBoost | Baoshan external | | 1539/243 | 0.832 (0.799–0.862) | | 0.588 (0.527–0.649) | 0.094 | 0.293 | 0.807 | | 1.076 (0.945–1.221) |  |
| **Panel B. External-validation differences: Fixed8 XGBoost minus reduced XGBoost** | | | | | | | | | | | | |
| **Reduced model** | | **ΔAUROC (95% CI)** | | | **P value** | | **ΔAUPRC (95% CI)** | | | **ΔBrier (95% CI)** | | |
| No-HbA1c XGBoost | | −0.003 (−0.009 to 0.003) | | | 0.402 | | 0.012 (−0.007 to 0.029) | | | 0.001 (−0.001 to 0.002) | | |
| No-HbA1c/fibrinogen/uric acid XGBoost | | −0.002 (−0.011 to 0.008) | | | 0.699 | | 0.003 (−0.015 to 0.022) | | | 0.000 (−0.002 to 0.002) | | |
| Routine4 XGBoost | | −0.013 (−0.024 to −0.002) | | | 0.021 | | −0.006 (−0.025 to 0.013) | | | 0.002 (0.000 to 0.004) | | |
| **Note:** Routine4 included age, blood urea nitrogen, baseline serum creatinine, and lymphocyte percentage. Positive ΔAUROC and ΔAUPRC values favor Fixed8 XGBoost; negative ΔBrier values favor Fixed8 XGBoost. All differences were estimated using 2000 paired bootstrap replicates. | | | | | | | | | | | | |

| **Supplementary Table S7. Complete-case and HbA1c-availability sensitivity analyses** | | | | | | | | | | |
| --- | --- | --- | --- | --- | --- | --- | --- | --- | --- | --- |
| **Panel A. Complete-case model performance** | | | | | | | | | | |
| **Model** | **Cohort** | **n/events** | **AUROC (95% CI)** | | **AUPRC (95% CI)** | **Brier** | **Scaled Brier** | **Calibration slope** | | **O/E (95% CI)** |
| Fixed8 logistic regression | Dali complete-case OOF | 1186/142 | 0.867 (0.834–0.898) | | 0.582 (0.508–0.665) | 0.073 | 0.307 | 1.025 | | 0.999 (0.841–1.177) |
| Fixed8 logistic regression | Baoshan complete-case external | 252/50 | 0.801 (0.723–0.871) | | 0.586 (0.471–0.715) | 0.124 | 0.221 | 0.761 | | 1.348 (1.001–1.778) |
| Fixed8 XGBoost | Dali complete-case OOF | 1186/142 | 0.871 (0.836–0.905) | | 0.537 (0.466–0.622) | 0.074 | 0.299 | 1.020 | | 1.030 (0.867–1.214) |
| Fixed8 XGBoost | Baoshan complete-case external | 252/50 | 0.805 (0.732–0.875) | | 0.607 (0.500–0.721) | 0.122 | 0.236 | 0.730 | | 1.375 (1.021–1.813) |
| **Panel B. CA-AKI frequency according to HbA1c availability** | | | | | | | | | | |
| **Cohort** | **HbA1c availability** | | | **n** | | **CA-AKI events** | | | **Event rate** | |
| Dali | HbA1c measured | | | 1492 | | 184 | | | 12.3% | |
| Dali | HbA1c missing | | | 1185 | | 118 | | | 10.0% | |
| Baoshan | HbA1c measured | | | 268 | | 56 | | | 20.9% | |
| Baoshan | HbA1c missing | | | 1271 | | 187 | | | 14.7% | |
| **Panel C. Selected baseline characteristics by HbA1c availability** | | | | | | | | | | |
| **Cohort** | **Characteristic** | | | **HbA1c measured** | | **HbA1c missing** | | | **SMD** | |
| Dali | Age, years | | | 60.0 (53.0, 70.0) | | 61.0 (53.0, 70.0) | | | 0.004 | |
| Dali | Blood urea nitrogen, mmol/L | | | 5.78 (4.61, 7.23) | | 5.80 (4.61, 7.05) | | | 0.077 | |
| Dali | Baseline serum creatinine, μmol/L | | | 76.0 (65.8, 93.0) | | 79.0 (67.0, 94.0) | | | 0.007 | |
| Dali | Fibrinogen, g/L | | | 3.35 (2.80, 4.20) | | 3.21 (2.70, 4.10) | | | 0.133 | |
| Dali | Lymphocyte percentage, % | | | 22.3 (15.6, 28.1) | | 23.0 (16.6, 29.5) | | | 0.077 | |
| Dali | Uric acid, μmol/L | | | 355.0 (290.0, 428.0) | | 364.0 (298.0, 440.0) | | | 0.035 | |
| Dali | eGFR, mL/min/1.73 m² | | | 94.1 (76.6, 103.9) | | 92.7 (76.1, 102.5) | | | 0.043 | |
| Dali | Diabetes mellitus, n (%) | | | 518 (34.7) | | 106 (8.9) | | | 0.657 | |
| Dali | RAS inhibitor use, n (%) | | | 490 (32.8) | | 326 (27.5) | | | 0.116 | |
| Baoshan | Age, years | | | 64.0 (55.0, 72.0) | | 66.0 (57.0, 73.0) | | | 0.089 | |
| Baoshan | Blood urea nitrogen, mmol/L | | | 5.73 (4.57, 7.71) | | 5.81 (4.67, 7.42) | | | 0.028 | |
| Baoshan | Baseline serum creatinine, μmol/L | | | 78.5 (63.6, 96.5) | | 75.0 (62.7, 91.9) | | | 0.033 | |
| Baoshan | Fibrinogen, g/L | | | 3.36 (2.92, 4.00) | | 3.32 (2.87, 3.94) | | | 0.082 | |
| Baoshan | Lymphocyte percentage, % | | | 18.7 (12.6, 25.6) | | 19.1 (12.6, 26.1) | | | 0.057 | |
| Baoshan | Uric acid, μmol/L | | | 350.5 (274.0, 431.6) | | 345.0 (277.0, 436.0) | | | 0.011 | |
| Baoshan | eGFR, mL/min/1.73 m² | | | 92.1 (69.9, 101.2) | | 91.7 (73.9, 101.5) | | | 0.053 | |
| Baoshan | Diabetes mellitus, n (%) | | | 146 (54.5) | | 142 (11.2) | | | 1.039 | |
| Baoshan | RAS inhibitor use, n (%) | | | 113 (42.2) | | 555 (43.7) | | | 0.030 | |
| **Note:** Complete-case analyses included patients with all eight predictors observed. Values in Panel C are median (interquartile range) or n (%). CA-AKI, contrast-associated acute kidney injury; HbA1c, glycated hemoglobin A1c; SMD, standardized mean difference. | | | | | | | | | | |

| **Supplementary Table S8. Age-stratified performance of the fixed eight-predictor XGBoost model** | | | | | | | | | | | | | | |
| --- | --- | --- | --- | --- | --- | --- | --- | --- | --- | --- | --- | --- | --- | --- |
| **Panel A. Discrimination and calibration** | | | | | | | | | | | | | | |
| **Cohort** | **Age group** | | **n/events (event rate)** | | **AUROC (95% CI)** | | **AUPRC (95% CI)** | | **Brier** | **Scaled Brier** | **Calibration slope** | | **O/E (95% CI)** |  |
| Dali OOF | <60 years | | 1236/91 (7.4%) | | 0.857 (0.808–0.900) | | 0.499 (0.400–0.605) | | 0.049 | 0.284 | 1.110 | | 0.965 (0.777–1.185) |  |
| Dali OOF | ≥60 years | | 1441/211 (14.6%) | | 0.875 (0.847–0.900) | | 0.595 (0.533–0.663) | | 0.083 | 0.334 | 0.990 | | 1.029 (0.895–1.178) |  |
| Baoshan external | <60 years | | 502/52 (10.4%) | | 0.763 (0.680–0.835) | | 0.415 (0.302–0.553) | | 0.078 | 0.159 | 0.708 | | 1.387 (1.036–1.819) |  |
| Baoshan external | ≥60 years | | 1036/191 (18.4%) | | 0.830 (0.794–0.865) | | 0.625 (0.561–0.690) | | 0.105 | 0.304 | 0.779 | | 0.995 (0.859–1.147) |  |
| **Panel B. Classification performance at the locked overall threshold** | | | | | | | | | | | | | | |
| **Cohort** | | **Age group** | | **Sensitivity** | | **Specificity** | | **PPV** | | **NPV** | **F1** | **Accuracy** | |  |
| Dali OOF | | <60 years | | 0.736 | | 0.866 | | 0.303 | | 0.976 | 0.429 | 0.856 | |  |
| Dali OOF | | ≥60 years | | 0.839 | | 0.756 | | 0.371 | | 0.965 | 0.515 | 0.768 | |  |
| Baoshan external | | <60 years | | 0.500 | | 0.898 | | 0.361 | | 0.940 | 0.419 | 0.857 | |  |
| Baoshan external | | ≥60 years | | 0.806 | | 0.684 | | 0.366 | | 0.940 | 0.503 | 0.707 | |  |
| **Panel C. Difference in AUROC between age groups** | | | | | | | | | | | | | | |
| **Cohort** | | | | **ΔAUROC: ≥60 minus <60 (95% CI)** | | | | **P value** | | | **Bootstrap replicates** | | |  |
| Dali OOF | | | | 0.017 (−0.035 to 0.076) | | | | 0.529 | | | 2000 | | |  |
| Baoshan external | | | | 0.067 (−0.014 to 0.156) | | | | 0.111 | | | 2000 | | |  |
| **Note:** The model, preprocessing, and classification threshold were applied without subgroup-specific refitting or optimization. AUPRC, area under the precision–recall curve; AUROC, area under the receiver operating characteristic curve; CI, confidence interval; NPV, negative predictive value; O/E, observed-to-expected ratio; OOF, out-of-fold; PPV, positive predictive value; XGBoost, extreme gradient boosting. | | | | | | | | | | | | | | |

| **Supplementary Table S9. Complete baseline characteristics of the derivation and geographical external validation cohorts** | | | |
| --- | --- | --- | --- |
| **Characteristic** | **Dali derivation cohort (n = 2677)** | **Baoshan external validation cohort (n = 1539)** | **SMD** |
| Demographics | | | |
| Age, years | 60.0 (53.0, 70.0) | 66.0 (57.0, 73.0) | 0.338 |
| Male sex, n (%) | 2110 (78.8) | 1048 (68.1) | 0.245 |
| Body mass index, kg/m² | 24.2 (23.3, 25.1) | 23.0 (20.6, 25.4) | 0.364 |
| Never smoker, n (%) | 1266 (47.3) | 438 (28.5) | 0.396 |
| Former smoker, n (%) | 161 (6.0) | 578 (37.6) | 0.827 |
| Current smoker, n (%) | 1250 (46.7) | 523 (34.0) | 0.261 |
| Current alcohol use, n (%) | 718 (26.8) | 314 (20.4) | 0.152 |
| Clinical presentation and comorbidities | | | |
| ST-segment elevation myocardial infarction, n (%) | 1743 (65.1) | 518 (33.7) | 0.663 |
| NSTE-ACS, n (%) | 934 (34.9) | 1021 (66.3) | 0.663 |
| Killip class | 1.0 (1.0, 2.0) | 1.0 (1.0, 2.0) | 0.415 |
| Hypertension, n (%) | 1410 (52.7) | 813 (52.8) | 0.003 |
| Diabetes mellitus, n (%) | 624 (23.3) | 288 (18.7) | 0.113 |
| Heart failure, n (%) | 2307 (86.2) | 952 (61.9) | 0.577 |
| Atrial fibrillation, n (%) | 115 (4.3) | 75 (4.9) | 0.028 |
| Cerebral infarction, n (%) | 171 (6.4) | 172 (11.2) | 0.170 |
| Peripheral artery disease, n (%) | 1164 (43.5) | 15 (1.0) | 1.189 |
| Anemia, n (%) | 95 (3.5) | 88 (5.7) | 0.103 |
| Infection, n (%) | 925 (34.6) | 184 (12.0) | 0.555 |
| Cancer, n (%) | 16 (0.6) | 20 (1.3) | 0.072 |
| Chronic lung disease, n (%) | 913 (34.1) | 189 (12.3) | 0.535 |
| Respiratory failure, n (%) | 56 (2.1) | 60 (3.9) | 0.106 |
| Renal function and laboratory variables | | | |
| Baseline serum creatinine, μmol/L | 77.0 (66.0, 93.0) | 75.3 (62.8, 92.5) | 0.003 |
| Estimated glomerular filtration rate, mL/min/1.73 m² | 93.5 (76.4, 103.2) | 91.7 (73.0, 101.5) | 0.129 |
| <30 mL/min/1.73 m², n (%) | 45 (1.7) | 41 (2.7) | 0.067 |
| 30–59 mL/min/1.73 m², n (%) | 235 (8.8) | 186 (12.1) | 0.108 |
| 60–89 mL/min/1.73 m², n (%) | 844 (31.5) | 493 (32.0) | 0.011 |
| ≥90 mL/min/1.73 m², n (%) | 1553 (58.0) | 818 (53.2) | 0.098 |
| Blood urea nitrogen, mmol/L | 5.79 (4.61, 7.15) | 5.81 (4.64, 7.46) | 0.094 |
| Uric acid, μmol/L | 359.0 (294.0, 433.0) | 346.0 (276.4, 434.5) | 0.053 |
| Glucose, mmol/L | 5.61 (4.96, 6.76) | 5.82 (5.01, 7.36) | 0.161 |
| Glycated hemoglobin A1c, % | 6.62 (6.13, 7.22) | 6.00 (5.50, 7.60) | 0.208 |
| Total cholesterol, mmol/L | 4.48 (3.73, 5.38) | 4.61 (3.89, 5.51) | 0.107 |
| Triglycerides, mmol/L | 1.64 (1.17, 2.42) | 1.34 (0.98, 2.04) | 0.136 |
| High-density lipoprotein cholesterol, mmol/L | 1.05 (0.88, 1.24) | 1.15 (0.96, 1.34) | 0.329 |
| Low-density lipoprotein cholesterol, mmol/L | 2.39 (1.81, 3.01) | 2.78 (2.14, 3.41) | 0.372 |
| Albumin, g/L | 37.7 (35.0, 40.2) | 38.7 (35.7, 41.7) | 0.241 |
| White blood cell count, ×10⁹/L | 7.69 (6.19, 9.94) | 7.84 (6.13, 10.23) | 0.060 |
| Neutrophil count, ×10⁹/L | 4.98 (3.70, 7.06) | 5.49 (3.97, 7.84) | 0.185 |
| Lymphocyte count, ×10⁹/L | 1.67 (1.28, 2.13) | 1.42 (1.06, 1.89) | 0.296 |
| Hemoglobin, g/L | 144.0 (132.0, 156.0) | 140.0 (129.0, 152.0) | 0.198 |
| Platelet count, ×10⁹/L | 211.0 (172.0, 255.0) | 200.0 (160.0, 241.0) | 0.234 |
| Mean corpuscular volume, fL | 91.5 (88.7, 94.5) | 91.8 (88.7, 95.2) | 0.014 |
| Neutrophil percentage, % | 65.7 (58.6, 73.8) | 71.5 (63.5, 80.0) | 0.470 |
| Lymphocyte percentage, % | 22.5 (16.0, 28.8) | 19.0 (12.6, 26.0) | 0.313 |
| Monocyte percentage, % | 8.0 (6.6, 9.5) | 6.4 (5.0, 8.0) | 0.615 |
| Hematocrit, % | 42.5 (39.3, 45.9) | 38.7 (33.5, 43.1) | 1.012 |
| Red cell distribution width–coefficient of variation, % | 13.1 (12.6, 13.7) | 13.4 (12.9, 14.2) | 0.370 |
| Potassium, mmol/L | 3.98 (3.70, 4.26) | 3.96 (3.70, 4.24) | 0.111 |
| Sodium, mmol/L | 141.0 (139.0, 143.0) | 141.5 (139.6, 143.2) | 0.017 |
| Chloride, mmol/L | 105.6 (103.5, 107.7) | 105.6 (102.7, 108.0) | 0.114 |
| Calcium, mmol/L | 2.18 (2.10, 2.26) | 2.20 (2.12, 2.28) | 0.059 |
| N-terminal pro-B-type natriuretic peptide, pg/mL | 643.5 (238.5, 1759.5) | 694.5 (172.8, 2599.0) | 0.251 |
| Alanine aminotransferase, U/L | 32.0 (20.0, 51.0) | 28.0 (20.0, 46.0) | 0.039 |
| Aspartate aminotransferase, U/L | 38.0 (23.0, 84.0) | 33.0 (23.8, 67.1) | 0.057 |
| Total bilirubin, μmol/L | 10.4 (7.7, 14.5) | 12.8 (9.2, 17.4) | 0.341 |
| Fibrinogen, g/L | 3.27 (2.75, 4.12) | 3.33 (2.88, 3.95) | 0.087 |
| Prothrombin time, s | 11.6 (10.6, 13.0) | 13.3 (12.6, 14.0) | 0.808 |
| Activated partial thromboplastin time, s | 29.9 (26.5, 35.6) | 38.2 (34.1, 53.3) | 0.763 |
| International normalized ratio | 0.96 (0.91, 1.02) | 1.05 (0.99, 1.12) | 0.506 |
| Echocardiographic variables | | | |
| Left ventricular ejection fraction, % | 65.0 (60.2, 70.0) | 65.0 (58.0, 72.0) | 0.061 |
| Left ventricular end-diastolic diameter, mm | 43.0 (41.0, 44.0) | 59.0 (47.0, 69.0) | 1.259 |
| Left atrial enlargement, n (%) | 490 (18.3) | 620 (40.3) | 0.498 |
| Left ventricular enlargement, n (%) | 406 (15.2) | 494 (32.1) | 0.407 |
| Pulmonary hypertension, n (%) | 290 (10.8) | 270 (17.5) | 0.193 |
| Medication use | | | |
| RAS inhibitor use, n (%) | 816 (30.5) | 668 (43.4) | 0.270 |
| β-blocker use, n (%) | 1952 (72.9) | 1200 (78.0) | 0.118 |
| Dihydropyridine calcium-channel blocker use, n (%) | 325 (12.1) | 163 (10.6) | 0.049 |
| Diuretic use, n (%) | 859 (32.1) | 134 (8.7) | 0.606 |
| Sodium–glucose cotransporter 2 inhibitor use, n (%) | 756 (28.2) | 183 (11.9) | 0.417 |
| Metformin use, n (%) | 127 (4.7) | 35 (2.3) | 0.135 |
| Thiazolidinedione use, n (%) | 4 (0.1) | 3 (0.2) | 0.011 |
| α-glucosidase inhibitor use, n (%) | 86 (3.2) | 39 (2.5) | 0.041 |
| Insulin use, n (%) | 92 (3.4) | 0 (0.0) | 0.267 |
| Outcome | | | |
| Contrast-associated acute kidney injury, n (%) | 302 (11.3) | 243 (15.8) | 0.132 |
| **Note:** Data are presented as median (interquartile range) for continuous variables and n (%) for categorical variables. Percentages use the full cohort denominator; detailed variable-level missingness is reported in Supplementary Table S2. SMD values are absolute standardized mean differences. NSTE-ACS includes NSTEMI and unstable angina because these subtypes could not be reliably distinguished in the source data. CA-AKI, contrast-associated acute kidney injury; eGFR, estimated glomerular filtration rate; HbA1c, glycated hemoglobin A1c; NSTE-ACS, non-ST-segment elevation acute coronary syndrome; NSTEMI, non-ST-segment elevation myocardial infarction; NT-proBNP, N-terminal pro-B-type natriuretic peptide; RAS, renin–angiotensin system; SMD, standardized mean difference; STEMI, ST-segment elevation myocardial infarction. | | | |

**Supplementary Figure S1. Age-stratified performance of the fixed eight-predictor XGBoost model in the geographical external validation cohort. A**, discrimination among patients aged <60 years and ≥60 years, assessed using the area under the receiver operating characteristic curve and the area under the precision–recall curve, with 95% confidence intervals. **B**, calibration in the same age groups, assessed using the observed-to-expected event ratio with 95% confidence intervals. The vertical dashed line indicates ideal overall calibration at an observed-to-expected event ratio of 1.0. The locked prediction model and preprocessing procedure developed in the Dali cohort were applied to both age groups without refitting, subgroup-specific model selection, or subgroup-specific recalibration. AUROC, area under the receiver operating characteristic curve; AUPRC, area under the precision–recall curve; CI, confidence interval; O/E, observed-to-expected event ratio; XGBoost, extreme gradient boosting.

**Supplementary Figure S2. SHAP dependence plots for the fixed eight predictors in the final XGBoost model.** Dependence plots show the relationship between each predictor value and its corresponding SHAP value for blood urea nitrogen, baseline serum creatinine, RAS inhibitor use, lymphocyte percentage, uric acid, fibrinogen, age, and HbA1c. Each point represents one participant in the derivation cohort. Positive SHAP values indicate contributions toward a higher model-predicted risk of CA-AKI, whereas negative values indicate contributions toward a lower predicted risk. The horizontal reference line at zero denotes no contribution relative to the model baseline output. The plots describe the behavior of the fitted prediction model and should not be interpreted as causal associations or clinically validated thresholds. CA-AKI, contrast-associated acute kidney injury; HbA1c, glycated hemoglobin A1c; RAS, renin–angiotensin system; SHAP, SHapley Additive exPlanations; XGBoost, extreme gradient boosting.

**Supplementary Figure S3. Partial dependence plots of the final fixed eight-predictor XGBoost model.** Partial dependence plots show the mean model-predicted probability of CA-AKI across the observed feature grid for age (**A**), blood urea nitrogen (**B**), baseline serum creatinine (**C**), fibrinogen (**D**), lymphocyte percentage (**E**), RAS inhibitor use (**F**), uric acid (**G**), and HbA1c (**H**), while averaging over the empirical distribution of the remaining predictors. RAS inhibitor use is presented as a binary predictor. The curves describe the average response of the fitted model and should not be interpreted as causal effects, exposure–response relationships, or clinically validated thresholds. CA-AKI, contrast-associated acute kidney injury; HbA1c, glycated hemoglobin A1c; RAS, renin–angiotensin system; XGBoost, extreme gradient boosting.
